# An Integrated Whole‐Process Repair System with Programmed Regulation of Healing Performance Facilitates Urethral Wound Restoration and Scarless Reconstruction

**DOI:** 10.1002/advs.202409930

**Published:** 2024-12-18

**Authors:** Wenzhuo Fang, Ying Wang, Kaile Zhang, Ming Yang, Meng Liu, Yangwang Jin, Xianjie Xiu, Yuhui Wang, Zhenwei Yu, Ranxing Yang, Qiang Fu

**Affiliations:** ^1^ Department of Urology Affiliated Sixth People's Hospital Shanghai Jiaotong University School of Medicine No. 600 Yi‐Shan Road Shanghai 200233 P. R. China

**Keywords:** hydrogel, scarless reconstruction, tissue engineering, urethral injury, whole‐process repair

## Abstract

The harsh microenvironment of the urethral injury often carries high risks of early undesirable healing as well as late scar tissue formation. Indeed, the infection and inflammatory response in the early stages as well as blood vessel formation and tissue regeneration in the later stages fundamentally impact the outcomes of urethral wound healing. Innovatively, an integrated whole‐process repair hydrogel (APF/C/L@dECM) is designed. After rigorous testing, it is found that hydrogels formed by hydrophobic association and double cross‐linking of amide bonds can procedurally regulate wound healing in all phases to match the repair process. In rabbit models of urethral wound, APF/C/L@dECM hydrogel can achieve scarless reconstruction with significantly better results than other hydrogels. Noteworthily, multi‐stage mechanistic explorations reveal the expression profiles of inflammation, vascularization, and extracellular matrix secretion‐related genes in wound tissue at different times. In summary, this study develops an overall treatment for urethral injury through a whole‐process repair system that promotes healthy healing of urethral wounds and prevents the formation of scar tissue.

## Graphical abstract

The whole‐process repair system (APF/C/L@dECM) synthesized ultimately achieves scarless reconstruction after urethral injury by promoting blood coagulation during the hemostasis phase, reducing inflammation during the inflammatory phase, and enhancing angiogenesis and matrix secretion during the proliferative phase of wound healing. It is expected to be used as an adjunctive treatment for urethral injuries in the future.

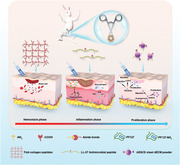

## 1 Introduction

Urethral injury is one of the most common injuries to the urinary system, with an increasing incidence in recent years.^[^ ^1^ ^]^ In clinical practice, patients with urethral injury are usually treated using indwelling catheterization, early endoscopic realignment, and suprapubic cystostomy (depending on the location and severity of the injury).^[^ ^2^ ^]^ However, these treatments are mainly aimed at restoring urethral continuity and patency and are not effective in wound recovery and tissue regeneration at the site of injury. The microenvironment of the urethra after injury is characterized by deep tissue wounds, bacterial infection, inflammation, poor vascularization, and ineffective tissue regeneration. Collectively, these aspects contribute to wound healing dysregulation. Improper urethral healing can contribute to abnormal cell proliferation, remodeling of the extracellular matrix (ECM), as well as an excessive myofibroblast accumulation. In contrast to the ECM of the normal urethra, urethral ECM with scar tissue formed under the influence of these factors often shows excessive collagen deposition and abnormal collagen ratios, reduced elastin, and excessive proteoglycan deposition and imbalance in the regulation of matrix metalloproteinases (MMPs), potentially causing urethral stricture disease (USD).^[^ ^3^ ^]^ Notably, although various treatment methods such as anastomotic urethroplasty^[^ ^4^ ^]^ and substitution urethroplasty^[^ ^5^ ^]^ are currently available in clinical practice for the treatment of USD and have achieved excellent outcomes, there remains a gap in effectively promoting urethral wound healing and preventing the formation of urethral stricture. Therefore, USD development represents a significant challenge in urology, as most patients who experience urethral injury will go on to develop USD, albeit at different timepoints post‐injury.^[^ ^6^ ^]^ In recent years, the advancement of tissue engineering technology has enabled the development of urethral injury therapy.^[^ ^7^ ^]^ However, these studies predominantly utilize urethral mucosal defect models, focusing on hydrogel dressings or patches at the injury site as alternatives to suturing.^[^ ^8^ ^]^ This type of modeling and treatment primarily simulates existing urethroplasty in clinical practice for scar tissue has already formed. By comparison, there is a significant lack of research on the in‐situ injection of hydrogels at the injury site to prevent or minimize the formation of urethral scar tissue. In addition, most of the studies to date have focused on a single stage of the healing process (e.g., the early inflammatory or late proliferative stages) rather than the entire process. Moreover, although some success has been achieved with each of the trialed therapeutic strategies, their general efficacy and clinical applicability remain to be investigated in the context of the multifactorial characteristics of the unfavorable local urinary microenvironment that develops after urethral injury.

**Scheme 1:**
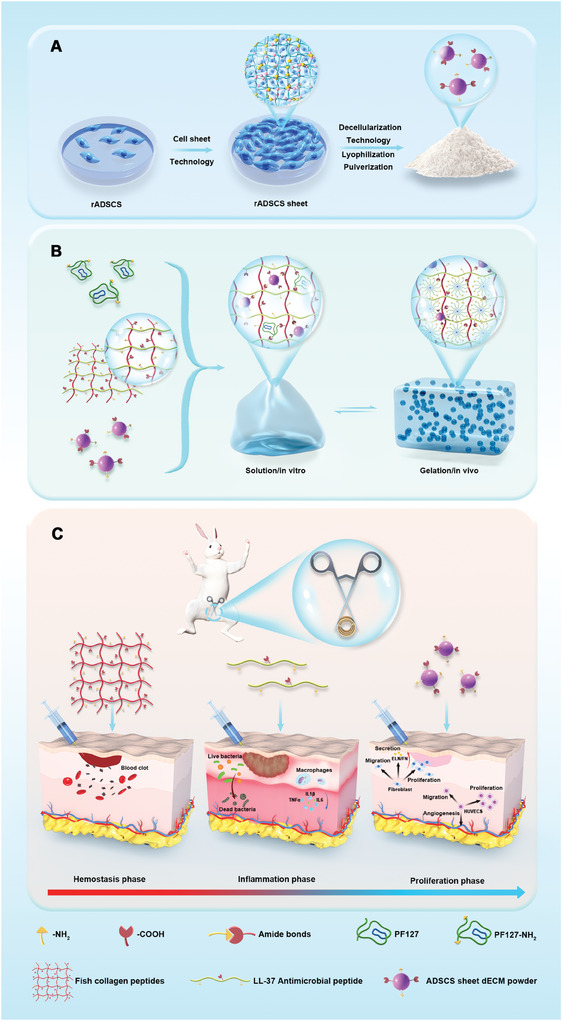
Schematic diagram of whole‐process repair hydrogel with programmed regulation of wound healing performance applied to rabbit urethral wound. A) Harvest of rADSCs sheet dECM powder. B) Construction of whole‐process repair hydrogel. C) programmed regulation of wound healing effects of rabbit urethral wound.

To overcome the above‐mentioned challenges, we designed a smart, whole‐process repair hydrogel with programmed regulation of wound healing performance as well as clinical translational potential for urethral wound repair (**Scheme** **1**). This approach aimed to restore the continuity and patency of the urethra after injury while locally injecting hydrogel in situ into the wound as an adjunctive treatment, which committed to efficiently and conveniently promote urethral wound healing in a way that prevent or minimize the formation of scar tissue in the later stages of wound healing. Therefore, in order to simulate the actual clinical situation of urethral trauma and iatrogenic injuries, we constructed a model of urethral injury in rabbits by clamping the urethra with hemostatic forceps to cause injury to the whole layers of urethral tissue.^[^ ^9^ ^]^ Our hydrogel is based on the Pluronic F‐127 (PF127) hydrogel, which is designed to be injectable and convenient for use in a clinical setting. PF127 is a temperature‐sensitive hydrogel with strong hydrophobic association and high biocompatibility, which has been approved for use in humans by the U.S. Food and Drug Administration (FDA).^[^ ^10^ ^]^ However, PF127 is unstable and easily degraded.^[^ ^11^ ^]^ To improve the stability of PF127, we grafted amino groups onto both ends of PF127 and introduced fish collagen peptides, as these natural polymers are commonly used in tissue repair and regeneration and have favorable hemostatic properties.^[^ ^12^ ^]^ We activated the carboxyl group of fish collagen peptides by 1‐Ethyl‐3‐(3‐dimethyl‐ aminopropyl‐1‐carbodiimide) (EDC)/ N‐hydroxysuccinimide (NHS), which allowed it to form amide bonds by combining with the amino group at the ends of PF127 and the amino group of collagens itself. Hydrophobic association and amide bond double crosslinking not only increase the stability of the hydrogel but also play a crucial role in the hemostatic phase of wound repair. A further challenge encountered during urethral injury treatment is the occurrence of severe local bacterial infections, which trigger scar‐promoting inflammatory reactions. Thus, the introduction of antibacterial and anti‐inflammatory agents early in the healing process is essential. To this end, we introduced the LL‐37 antibacterial peptide into the hydrogel system, to act during the inflammatory stage of the wound healing process.^[^ ^13^ ^]^ Angiogenesis, cell proliferation, and matrix secretion dominate the next, proliferative phase of wound healing. These processes require considerable amounts of growth factors and nutrients, which can be supplied by adipose‐derived stem cells (ADSCs).^[^ ^14^ ^]^ However, simply injecting stem cells is often inadequate. The harsh local microenvironment of the urethra can reduce cell viability and limit the cells' ability to secrete the nutrients required for wound healing. Furthermore, genetic differences between the recipient and the donor‐derived stem cells can lead to immune rejection, potentially impeding the wound‐healing process.^[^ ^15^ ^]^ We overcame this challenge by employing cell sheet technology and decellularization techniques to obtain decellularized (d)ECM from ADSC sheets. The ADSC sheets contain various cytokines and ECM components, such as sulfated glycosaminoglycans (GAGs), which not only promote wound healing but also possess anti‐inflammatory and antioxidant properties.^[^ ^16^ ^]^ The decellularization techniques were then used to selectively eliminated any immunogenic cellular components from the ADSC sheets while preserving the nutrient‐rich, ADSC‐derived ECM. This breakthrough offers significant potential for the large‐scale production and commercialization of ADSCs sheet dECM.^[^ ^17^ ^]^

In summary, the repair of urethral injury is a complex and challenging process, where improperly managed early wound healing can lead to subsequent scar tissue formation. In the present study, we developed a hydrogel capable of orchestrating effective healing and regeneration. In the initial phase of wound healing (the hemostasis phase), the collagen component of our hydrogel system promoted blood coagulation and stabilized the wound. During the second, inflammatory phase, the LL‐37 antimicrobial peptide not only provided antibacterial activity but also modulated immune and inflammatory mediators, creating a favorable immune microenvironment for tissue repair. In the third, proliferation phase, the ADSCs sheets dECM, which is enriched in various growth factors and nutrients, synergistically promoted tissue regeneration and angiogenesis. The three phases of wound healing are interconnected and overlapping. Our whole‐process repair hydrogel with programmed regulation of wound healing performance was capable of modulating healing at one or multiple stages to guide the entire tissue repair process. Histological and functional assessments revealed that this system effectively prevented the formation of late‐stage scar tissue and urethral stricture. Through RNA sequencing, quantitative real‐time polymerase chain reaction (qRT‐PCR), and western blotting (WB) analyses, conducted at various timepoints after treatment initiation, we observed that the upregulation of genes implicated in ECM secretion (e.g., *FN1* and *ELN*) and vascularization (e.g., *CD31* and *eNOS*), coupled with the downregulation of those involved in inflammation (e.g., *CD14*, *IL1β*, and *CD86*), was key to proper urethral wound healing and the prevention or minimization of late‐stage scar tissue formation. Collectively, our findings highlight the value of designing therapeutic agents that target every aspect of the tissue repair process.

## 2 Results

### 2.1 Characterization of the Integrated Whole‐Process Repair System

**Figure 1:**
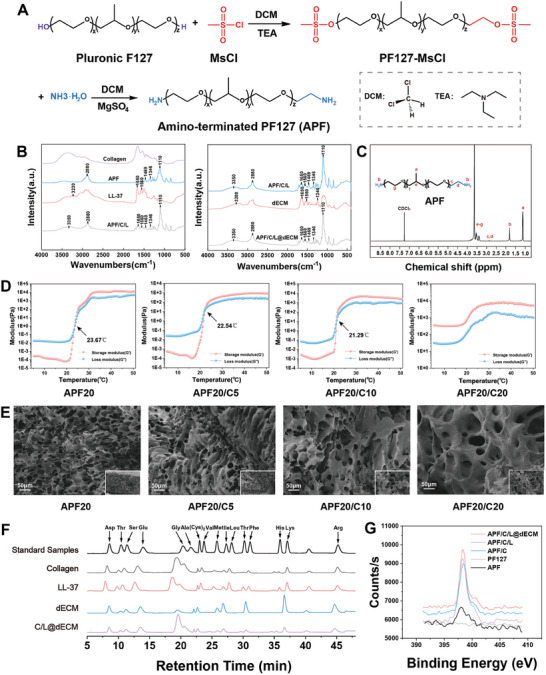
Generation and characterization of hydrogel. A) schematic diagram showing the process used to synthesize the APF polymer. B) FTIR spectra of the hydrogel components. C) ^1^H NMR spectra of the APF polymer in CDCl_3_. D) Temperature‐dependent rheology of hydrogels at 5–50 °C (G’, the storage modulus; G’’, the loss modulus). E) Vertical SEM images of APF20, APF20/C5, APF20/C10, and APF20/C20 hydrogels. The scale bar in both the high and low magnification images is 50 µm. F) The amino acid composition of the hydrogel components was determined using acid hydrolysis and ion chromatography. G) Peak‐fitting of the N1s regions XPS spectra of the surfaces of different hydrogels.

In this study, we used the PF127 hydrogel as a carrier. However, the in vitro degradation rate assays (phosphate‐buffered saline (PBS) and urine) revealed that PF127 was unstable and rapidly degraded (Figure S1, Supporting Information).^[^ ^18^ ^]^ To prolong the time for the hydrogel to function, we generated an amino‐terminated PF127 (APF) (**Figure** **1**A) and chemically crosslinked it with fish collagen peptides, which have procoagulant properties. It was found that the stability of the hydrogel was greatly improved for sustained efficacy (Figure S1, Supporting Information). In addition, we also found that the degradation of hydrogels was promoted in the urine environment. We used ^1^H nuclear magnetic resonance (NMR) to confirm that the end of PF127 had been successfully modified by the inclusion of amino groups (Figure 1C). Specifically, APF spectrum had new peaks at both 1.8 and 2.8 ppm, which were not present in the PF127 spectrum (Figure S2, Supporting Information). Furthermore, since the temperature of the normal rabbit urethra is ≈35 °C (Figure S3, Supporting Information), a system in which the hydrogel remained a liquid at room temperature and formed a gel at 35 °C was selected. Rheological testing revealed that 10% (by weight [wt.]) of APF could not turn into gel in rabbits, while 30% wt. of APF turned into gel at room temperature, which made it difficult to inject (Figure S4, Supporting Information). Therefore, we chose 20% wt. APF (APF20) for subsequent experiments. Next, we explored the change in modulus with temperature change. Different concentrations of collagens (5%, 10%, and 20% wt.) were crosslinked with APF20 (named APF20/C5, APF20/C10, and APF20/C20, respectively) under 2% wt. EDC/NHS activation, aiming to determine the optimal ratio of the components (APF20, APF20/C5, APF20/C10, APF20/C20). We found that the phase inversion temperature of the hydrogel decreased with increasing collagen concentration, generating a result that was consistent with the differential scanning calorimetry (DSC) curve (Figure 1D; Figure S5, Supporting Information). However, when the collagen concentration rose to 20% wt., the hydrogel solidified at room temperature, indicating that this concentration of collagen‐induced premature gel formation following EDC/NHS‐mediated activation.

We next evaluated the ultrastructure of the hydrogels formed under different conditions using scanning electron microscopy (SEM). We found that the interior of all hydrogels had an interoperable porous structure (Figure 1E). Raising the collagen concentration within the hydrogel increased the pore size and the thickness of the pore walls; however, it decreased the overall number of pores. In addition to the characterization of APF/C, we also used Fourier transform infrared (FTIR) to characterize the chemical structures of different components within the hydrogel (i.e., LL‐37, collagen, and dECM) (Figure 1B). Within the FTIR spectrum, 3350 and 3230 cm^−1^ indicated O─H stretching vibration, 1650 cm^−1^ indicated C≐O stretching vibration and 1560 cm^−1^ indicated the N─H bending vibration and C─N stretching vibration. The characteristic bands of APF were located at 2880, 1469, 1346, and 1110 cm^−1^, which represent C─H stretching, CH2 bending, in‐plane O─H bending, and C─O stretching, respectively. We also analyzed the amino acid composition of LL‐37, collagen, and dECM by acid hydrolysis and ion chromatography (Figure 1F). By comparing the X‐ray photoelectron spectroscopy (XPS) curve of the N1s fine spectrum curves of PF127 and APF we also found that the addition of the terminal amino group changed the composition of PF127 (Figure 1G).

### 2.2 Evaluation of Hydrogel Biocompatibility and Hemostatic and Antimicrobial Properties

**Figure 2:**
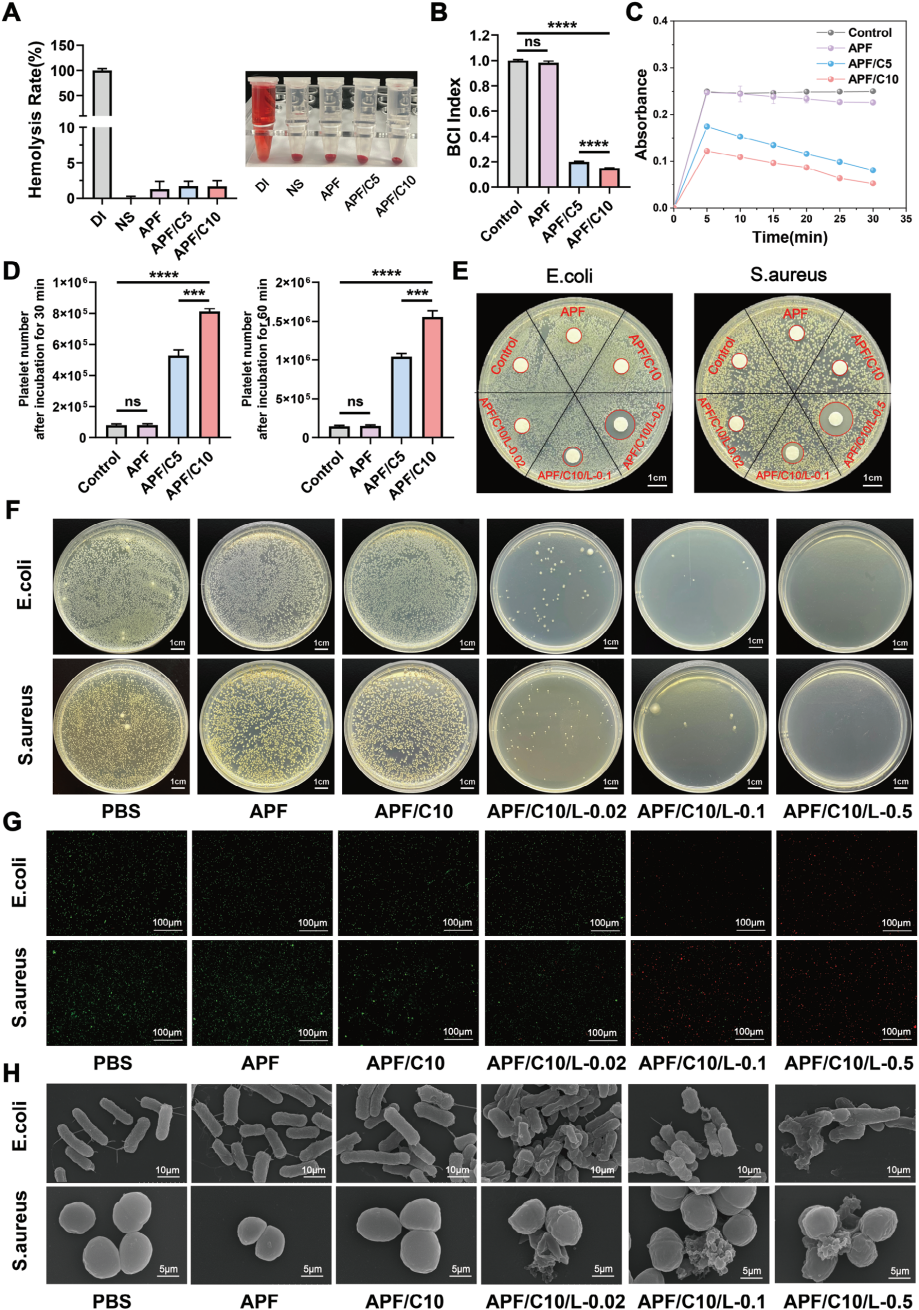
Evaluation of hydrogel biocompatibility and its procoagulant and antimicrobial properties in vitro. A) Hemolysis test results (*n* = 3 per group). B) BCI comparison of different hydrogels. C) Dynamic coagulation curves of the different hydrogels. D) Platelet number on different hydrogels after incubation for 30 and 60 min. E) Measurement of the zone of inhibition. F) Colonization. G) Bacterial live‐dead staining. The scale bar in images is 100 µm. H) SEM images of *S. aureus* and *E. coli* after treatment with PBS, APF, APF/C10, APF/C10/L‐0.02, APF/C10/L‐0.1, or APF/C10/L‐0.5. The scale bar in images is 5 or 10 µm. Data are expressed as the mean ± standard deviation (SD) (*n* = 3). ns, not significant (*p* > 0.05), ***p < 0.001, ****p < 0.0001.

In the harsh local microenvironment that forms following urethral injury, a hydrogel with prompt hemostatic capabilities and excellent antibacterial properties is highly desirable. To assess the biocompatibility of the hydrogel, we conducted a hemolysis test, which is commonly used to evaluate the quality of hemostatic materials. Typically, materials with a hemolysis rate of <5% meet the clinical blood safety requirements. The semi‐quantitative analysis confirmed that the hemolysis rates of all the hydrogels were below 5% (**Figure** **2**A; Figure S6A, Supporting Information), indicating that they had favorable biocompatibility and met the blood safety requirements.

During hemostasis, it is inevitable that some of the blood cannot be completely coagulated by the hemostatic material. We added distilled water into centrifuge tubes to obtain a hemoglobin solution, which was then used to evaluate the hemostatic performance of each hydrogel preparation. We determined the blood clotting index (BCI) of various materials by measuring their optical density (OD) values within the hemoglobin solution. The lower the BCI index, the better the coagulation effect of the material. The BCI indexes of APF, APF/C5, and APF/C10 were 0.98, 0.2, and 0.15, respectively, indicating that the hemostatic ability of the hydrogel increased with collagen concentration (Figure 2B; Figure S6B, Supporting Information). The absorbance change curves of the three materials at different timepoints (dynamic coagulation curves) confirmed that the APF/C10 hydrogel exhibited excellent hemostatic properties (Figure 2C; Figure S6C, Supporting Information). In order to assess whether the hydrogel would have an effect on platelet adhesion upon contact with blood, we performed platelet adhesion experiments. The results showed the same trend, where the addition of collagen improved the platelet adhesion properties of the hydrogels, which further exerted procoagulant properties (Figure 2D; Figure S6D, Supporting Information). Therefore, APF20/C10 was chosen for subsequent experiments.

Subsequently, LL‐37 antimicrobial peptide was introduced into the APF20/C10 hydrogel, which sparked our interest in evaluating its antimicrobial ability. Using *Staphylococcus aureus* (*S. aureus*) and *Escherichia coli* (*E. coli*) as bacterial models, we demonstrated that the increase in the size of the inhibition zone correlated positively with LL‐37 concentration within the hydrogel (Figure 2E); APF/C10/L‐0.02, APF/C10/L‐0.1, and APF/C10/L‐0.5 represent 0.02%, 0.1%, and 0.5% wt. LL‐37 concentrations in the hydrogel, respectively. Next, the bacterial suspensions treated with the different hydrogels were evenly spread onto bacterial culture plates. After 24 h, a visible decrease in bacterial counts was observed (Figure 2F; Figure S7A, Supporting Information). The results of the bacterial live/dead staining also followed a similar trend. The *S. aureus* and *E. coli* treated with PBS, APF, or APF/C10 exhibited significant green fluorescence, while introducing increasing concentrations of LL‐37 into the culture system strengthened the red fluorescent signal, indicating LL‐37‐induced bacterial eradication (Figure 2G; Figures S7B and S8, Supporting Information). To further determine the bactericidal effect of the hydrogel, we also used SEM to observe the morphological changes experienced by *S. aureus* and *E. coli* after exposure to the different hydrogel formats. *S. aureus* and *E. coli* treated with PBS, APF, or APF/C10 exhibited normal morphology, with intact and smooth cell membranes, indicating negligible damage to the bacteria (Figure 2H; Figure S7C, Supporting Information). On addition of LL‐37, however, the bacterial cell membranes appeared wrinkled and shrunken. When the LL‐37 concentration was increased to 0.5% wt., the bacterial cell membranes were completely obliterated, causing bacterial deformation and rupture. These results confirm that the APF/C10/L‐0.5 hydrogel exerts a potent bactericidal effect. All of these results demonstrated that 0.5% wt. of LL‐37 was sufficient to show satisfactory antimicrobial performance in vitro, and in consideration of factors such as production cost and time, we finally chose this system, APF/C10/L‐0.5 hydrogel (APF/C/L from hereon), for the subsequent experiments. We also further tested the release characteristics of hydrogels loaded with this concentration of LL‐37 antimicrobial peptide, and we found that the release of LL‐37 was basically in line with the trend of the degradation rate of the hydrogels, but slightly higher than the degradation rate, which suggests that the cross‐linking network of the hydrogel realizes a slow release of the LL‐37 antimicrobial peptide, and contributes to the sustained provision of an effective antimicrobial effect of the hydrogel (Figure S9, Supporting Information).

### 2.3 Characterization of ADSC Sheets and the Evaluation of their Biological Functions

**Figure 3:**
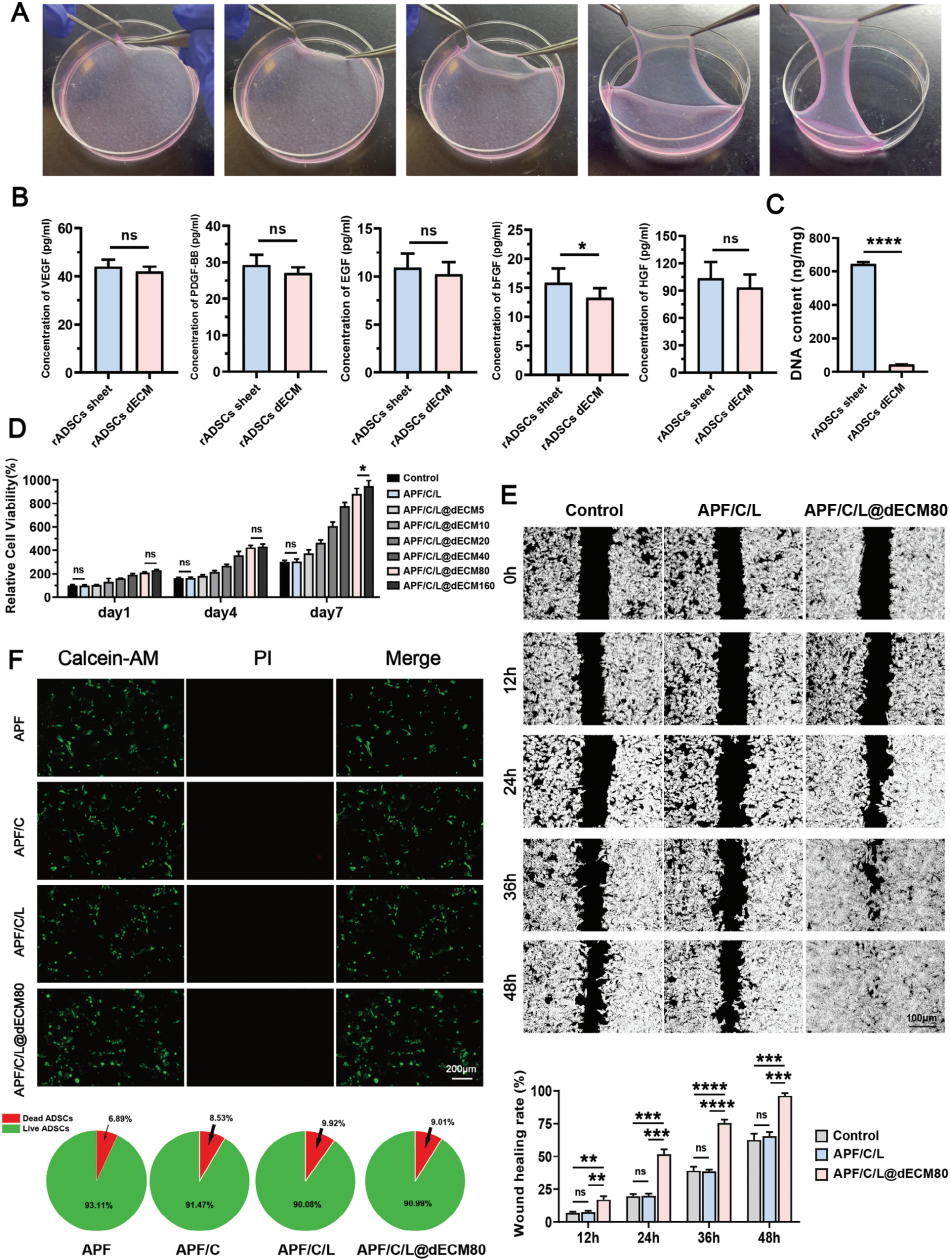
Characterization of cell sheets before/after decellularization and evaluation of the ability of ADSC dECM to promote cell proliferation and migration in vitro. A) ADSC sheets were successfully isolated from cultured cells. B) Concentrations of VEGF, PDGF‐BB, EGF, bFGF, and HGF in ADSCs sheets and ADSCs dECM, as determined by ELISA. C) DNA contents before/after decellularization. D) The cell proliferation rates of ADSCs at 1, 4, and 7 days after treatment were detected using the CCK‐8 assay. Data are expressed as the mean ± standard deviation (SD) (*n* = 6). E) Dark‐field images (and corresponding quantitative data obtained) of ADSCs cultured under different conditions (PBS, APF20/C10‐L‐0.5 [APF/C/L], and APF20/C10‐L‐0.5@dECM 80 [APF/C/L@dECM]) for 12, 24, 36, or 48 h in a wound healing assay; the cell‐free area was measured using ImageJ software. F) Fluorescence microscopy images and semi‐quantitative data showing live (green) and dead (red) cells in different treatment groups after calcein‐AM (green) and propidium iodide (PI, red) staining, respectively. ns, not significant (*p* > 0.05), *p < 0.05, **p < 0.01, ***p < 0.001, ****p < 0.0001.

Cell sheets are membranous materials formed by the aggregation and secretion of ECM components by cells; they are enriched in growth factors, which can provide nutrients for wound repair and regeneration. Intact ADSC sheets can be obtained from culture dishes using forceps (**Figure** **3**A). Using inverted microscopy, we observed that ADSCs cultured in ascorbic‐acid‐containing medium for 14 days assumed a highly fused state. Transmission electron microscopy (TEM) revealed the presence of numerous tight and gap junctions between cells, indicative of extensive substance exchange (Figure S10, Supporting Information). Subsequent decellularization of ADSC sheets significantly reduced their cellular deoxyribonucleic acid (DNA) content and immunogenicity. Although the concentration of basic fibroblast growth factor (bFGF) in the sheets decreased significantly after decellularization, there were no significant changes in the levels of vascular endothelial growth factor (VEGF), Platelet‐derived growth factor BB (PDGF‐BB), epidermal growth factor (EGF), bFGF, and hepatocyte growth factor (HGF) (Figure 3B,C). In fact, the concentrations of collagen and GAG actually increased slightly after the removal of the cellular components (Figure S11, Supporting Information). Hematoxylin and eosin stains (H&E), 4′,6‐diamidino‐2‐phenylindole (DAPI), Alcian blue, and Sirius Red staining before and after decellularization demonstrated that the process effectively removed nuclear material without disrupting the original tissue structure or the amounts of collagen and GAG (Figure S12, Supporting Information).

Given that cell sheets contain vast amounts of nutrients and growth factors, they can be used as powerful tools for boosting cell replication, migration, and proliferation. To incorporate ADSC dECM into the hydrogel scaffold, they were lyophilized, ground into powder, and added to the hydrogel at different concentrations (5, 10, 20, 40, 80, 160 mg mL^−1^). The optimal concentration of ADSC sheets dECM for promoting stem cell replication and proliferation was then determined. For clarity the APF/C10, APF/C10/L‐0.5, and APF/C10/L‐0.5@dECM5–160 hydrogels were named APF/C, APF/C/L, and APF/C/L@dECM5–160, respectively. By using ADSCs cells as the model, Cell Counting Kit‐8 (CCK8) results showed that APF/C/L hydrogel exhibited good biocompatibility; moreover, when the concentration of ADSC dECM within the hydrogel was increased to 80 mg mL^−1^, the hydrogel's ability to promote cell proliferation stabilized (Figure 3D). Subsequently, the APF/C/L@dECM‐80 hydrogel was subjected to rheological testing and met the requirements for injection (Figure S13, Supporting Information). Therefore, we chose the hydrogel containing 80 mg mL^−1^ dECM for subsequent experiments. In addition, we confirmed the ability of dECM powder to promote cell migration in a wound‐healing assay. Specifically, we showed that the ADSCs treated with APF/C/L@dECM‐80 covered ≈100% of the wound at 48 h, while the wound coverage rates of other treatment groups were <75% (Figure 3E; Figure S14, Supporting Information). Live/dead staining further confirmed the high biocompatibility of hydrogels and the ability of dECM powder to enhance cell survival (Figure 3F). After determining the final composition of each group of hydrogels, we examined the Young's modulus and mechanical compression properties of the hydrogels, and the results showed that collagen and dECM powder improved the stability and durability of the hydrogels (Figure S15, Supporting Information). However, the swelling rate assay showed that the swelling rate of the hydrogel decreased with the mixing of dECM powder, which could be attributed to the fact that the cross‐linking of the hydrogel became dense and the pore structure was significantly reduced by dECM (Figure S16, Supporting Information). Moreover, considering the dynamic and liquid environment of the urethra, rapid gel formation in vivo is necessary. Since hydrogels are injected in situ inside the tissues surrounding the injury, it is relatively difficult to assess the hydrogel solution‐gel transition time in vivo. Thus, we assessed the gelation time of different groups of hydrogels in vitro by visual observation of hydrogels by the tilted tube method. We found that as the gelation time was gradually shortened with the addition of collagen and dECM powder, the APF/C/L@dECM‐80 group even presented gelation at 45 s (Figure S17, Supporting Information).

### 2.4 In Vivo Urethral Wound Restoration and Scarless Reconstruction in Rabbits

**Figure 4:**
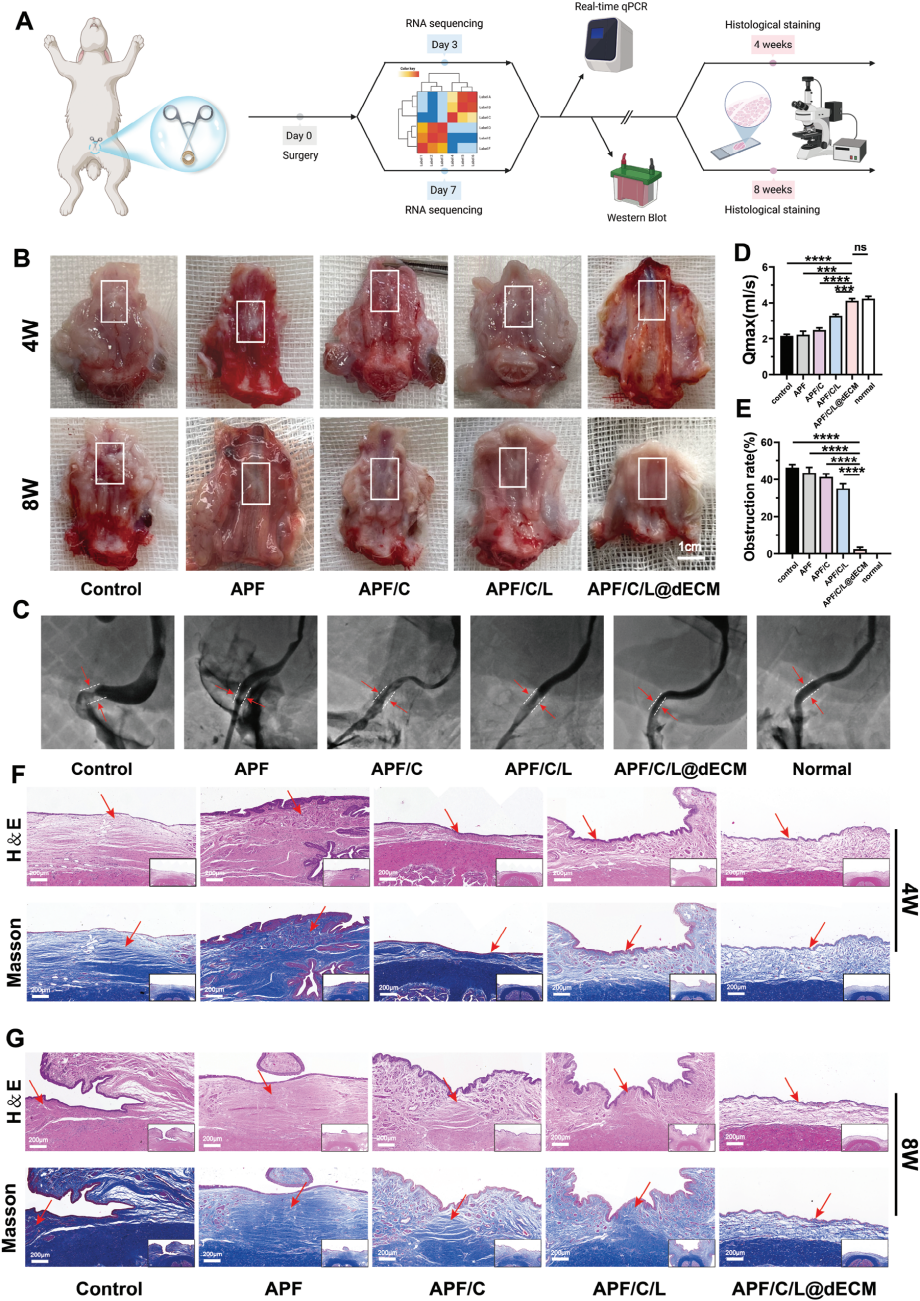
The whole‐process repair hydrogel achieves scarless urethral wound repair in rabbits. A) Timeline of the whole‐process repair hydrogel treatment of urethral injury in rabbits. B) Digital photographs of the extent of in vivo tissue regeneration at the site of injury at different time intervals after injection of the different hydrogel preparations near the wound site. Urethrography images C), Qmax values D), and urethra blockage rates E) of the rabbit urethra in the control (saline injection), APF, APF/C, APF/C/L, APF/C/L@dECM, and normal groups; the blockage rates were measured and analyzed using ImageJ software. Data are expressed as the mean ± standard deviation (SD) (*n* = 3). Pathological examination of rabbit urethra was performed after injection of different hydrogel preparations. Light microscopy images of H&E staining and Masson's staining after 4 weeks F) or 8 weeks G) post‐injury. The inset shows a low‐magnification image of the folds. The scale bar in both the high and low magnification images is 200 µm. ns, not significant (*p* > 0.05), ***p < 0.001, ****p < 0.0001.

To further evaluate the reparative effect of this whole‐process repair hydrogel with programmed regulation of wound healing performance in vivo, we constructed a rabbit urethral injury model. By clamping the urethra with hemostatic forceps, we caused whole‐layer tissue injury of the urethra to simulate the actual situation of patients with urethral injuries in the clinic (Figure S18, Supporting Information). In the following, different groups of hydrogels‐APF20 (APF), APF20/C10 (APF/C), APF20/C10‐L‐0.5 (APF/C/L), and APF20/C10‐L‐0.5@ dECM80 (APF/C/L@dECM) were injected in situ within the epithelial tissue at the injury site and at the surrounding connective tissue. We have drawn a timeline on the application of the whole‐process repair hydrogel in a rabbit model of urethral injury and methods for assessing its efficacy (**Figure** **4**A). Since the final assessment of good wound healing and scar formation requires waiting for the wound recovery to reach a mature stage with gradual stabilization of local cell and tissue metabolism, tissue harvesting is usually chosen to be performed at 4 and 8 W postoperatively. Assessment of gross morphology revealed that the control, APF, and APF/C groups all had significant scar tissue at 4 weeks after urethral injury induction, which subsequently led to USD development (Figure 4B). The rabbits treated by APF/C/L hydrogel exhibited a relatively smaller scar area, possibly due to the early anti‐inflammatory effects of LL‐37 incorporated into this preparation. The repair effect of APF/C/L@dECM group on the urethra was significantly superior to that of the other groups, as evidenced by a smooth and flat urethra, without signs of proliferative scar formation. The rabbits were subsequently subjected to urethrography to evaluate the extent of urethral tissue regeneration.^[^ ^19^ ^]^ The results showed that the rabbits in the control, APF, and APF/C groups had severely narrowed lumens due to scar hyperplasia, while those in the APF/C/L group had wider lumens but still exhibited a certain degree of obstruction (Figure 4C). In comparison with the other four experimental groups, the APF/C/L@dECM group had the lowest obstruction rate and the highest maximum flow rate (Qmax), which approached that of the normal group (Figure 4D,E). These results indicate that treatment with APF/C/L@dECM hydrogel healed the urethral wound without scar or fistula formation. Importantly, the newly generated tissue seamlessly integrated with the adjacent normal tissue, without any apparent boundaries. Of note, there was no obvious evidence of implant reaction or infection during specimen collection, and the rabbits remained healthy throughout the process.

Histological staining showed that the urethra healed poorly in rabbits belonging to the control, APF, and APF/C groups. Specifically, the wound structure was disorganized, with extensive fibroblast infiltration, high levels of local collagen fiber deposition, and thick proliferative scar tissue formed at 4‐ and 8‐weeks post‐injury (Figure 4F,G; Figure S19, Supporting Information). This suggests that APF and collagen alone do not have the efficacy to promote wound healing and that poor wound healing leads to the formation of scar tissue, as in most actual clinical cases. In line with the gross morphology findings, the rabbits in the APF/C/L group formed less scar tissue at 4 weeks post‐injury, but still developed scarring at 8 weeks; moreover, Masson staining indicated that there was obvious local collagen deposition in these animals. We next performed Sirius Red staining at the site of injury. Sirius Red, which exhibits birefringence when exposed to polarized light, can be used to distinguish between different types of collagen fiber. In general, type I collagen fibers are dense, firm, and orange/red in color, while type III collagen fibers are looser and appear green.^[^ ^20^ ^]^ Scar tissues have a higher type I to type III collagen ratio, causing them to appear orange following Sirius Red staining (Figure S20, Supporting Information). By contrast, the urethral tissue of rabbits in the APF/C/L@dECM group appeared green under polarized light following Sirius Red staining, was sparsely organized and structurally normal, with local cells and collagen fiber networks arranged in an orderly manner without any obvious signs of an inflammatory reaction. All of the above results confirm that the APF/C/L@dECM hydrogel enabled proper urethral wound healing and prevented the formation of scar tissue.

**Figure 5:**
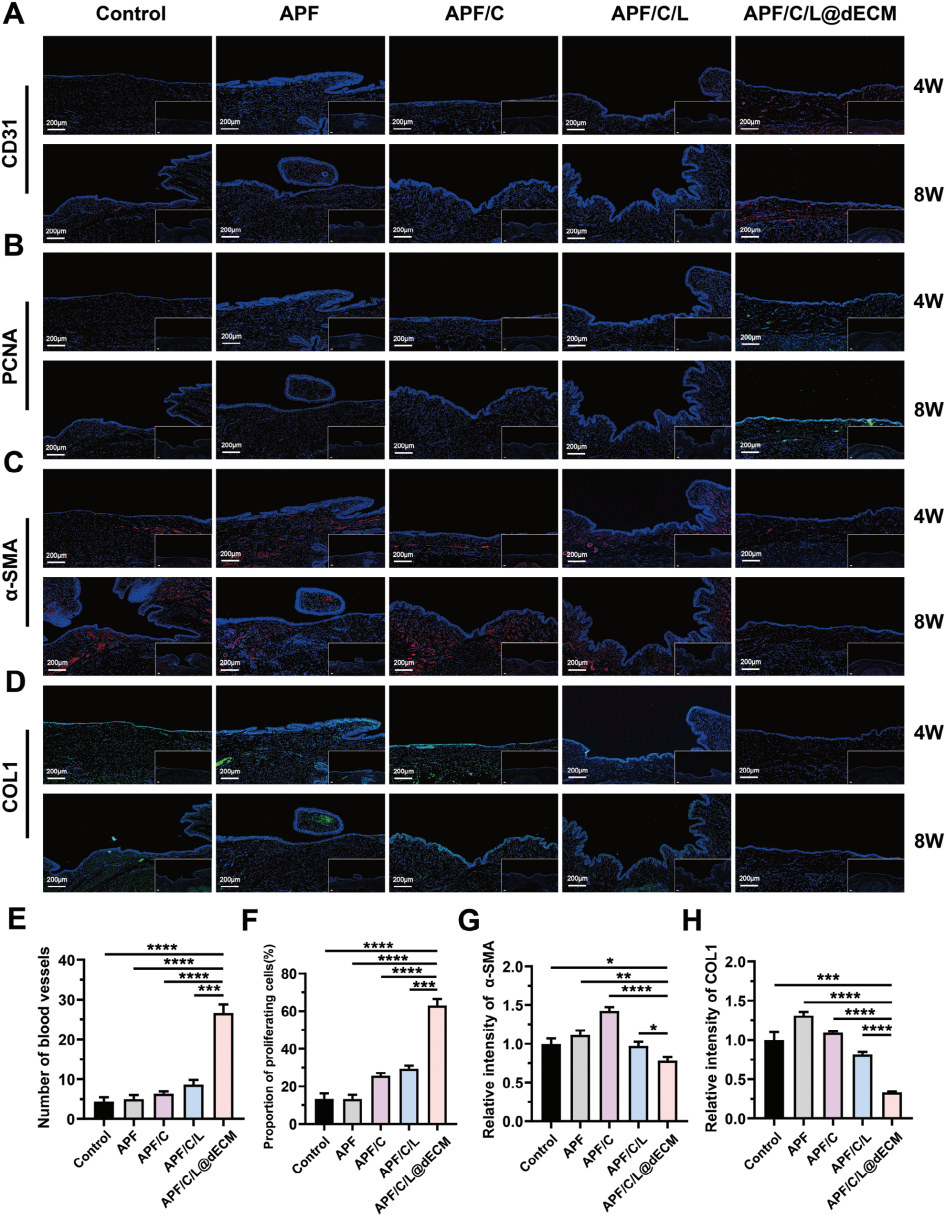
Immunofluorescence staining of tissue sections to examine the extent of scarring during urethral wound repair. Immunofluorescence images of angiogenesis (CD31) A), cell proliferation (PCNA) B), and fibrosis C,D); blue represents the nucleus, red (CD31/α‐SMA) and green (PCNA/COL1) represent the corresponding markers. The inset is the same area imaged at low magnification. The scale bar in both the high and low magnification images is 200 µm. Analysis of the number of vessels E), proportion of proliferating cells F), and relative fluorescence intensity of α‐SMA G) and COL1 H) in the urethral wounds were used to assess angiogenesis, cell proliferation, and fibrosis progression after 4 weeks of treatment with (i.e., control, APF, APF/C, APF/C/L, and APF/C/L@dECM). Data are expressed as the mean ± standard deviation (SD) (*n* = 3). *p < 0.05, **p < 0.01, ***p < 0.001, ****p < 0.0001.

To further understand how the whole‐process repair system regulated wound healing and achieved scarless urethral reconstruction in rabbits, we used immunofluorescence staining to monitor the expression of platelet endothelial cell adhesion molecule‐1 (CD31), proliferating cell nuclear antigen (PCNA), alpha‐smooth muscle actin (α‐SMA), and collagen 1 (COL1) (markers of vascular generation, cell proliferation, and fibrosis). All but the APF/C/L@dECM group exhibited low rates of vascularization and had a lower proportion of proliferative cells, which led to poor tissue regeneration and eventual scar formation (**Figure** **5**A,B,E,F). We found that the levels of α‐SMA and COL1 were high in the first four experimental groups, likely due to excessive fibroblast proliferation, collagen fiber deposition, and scar tissue formation at the injury site (Figure 5C,D). In addition, because the APF/C/L@dECM group exhibited high levels of angiogenesis its expression of α‐SMA was only slightly decreased (Figure 5G). However, the newly generated tissue was loose, orderly, and contained a lower proportion of type I collagen fibers, which aligned with the reduced fluorescence intensities of the corresponding markers (Figure 5H). As is well known, epithelial tissue regeneration is essential for urethral repair. Therefore, we evaluated the epithelial tissue regeneration in each group following injury repair by performing cytokeratin (AE1/AE3) immunofluorescence staining (Figure S21, Supporting Information). Given that we utilized a full‐thickness tissue injury model rather than mucosal defect models, the control group, while displaying some epithelial loss, still showed a degree of coverage. Compared to the other groups, the APF/C/L@dECM group exhibited significantly enhanced epithelial regeneration, which may be attributed to the growth factors and nutrients enriched in dECM powder that promote epithelial cell proliferation and migration, thereby greatly enhancing urethral injury healing and tissue repair capabilities. Overall, these data demonstrate that APF/C/L@dECM promotes the scarless wound healing of urethral tissue by facilitating angiogenesis and cell proliferation.

### 2.5 Molecular Mechanisms Underlying the Ability of the Whole‐Process Repair System to Promote Wound Healing and Prevent Scar Tissue Formation

**Figure 6:**
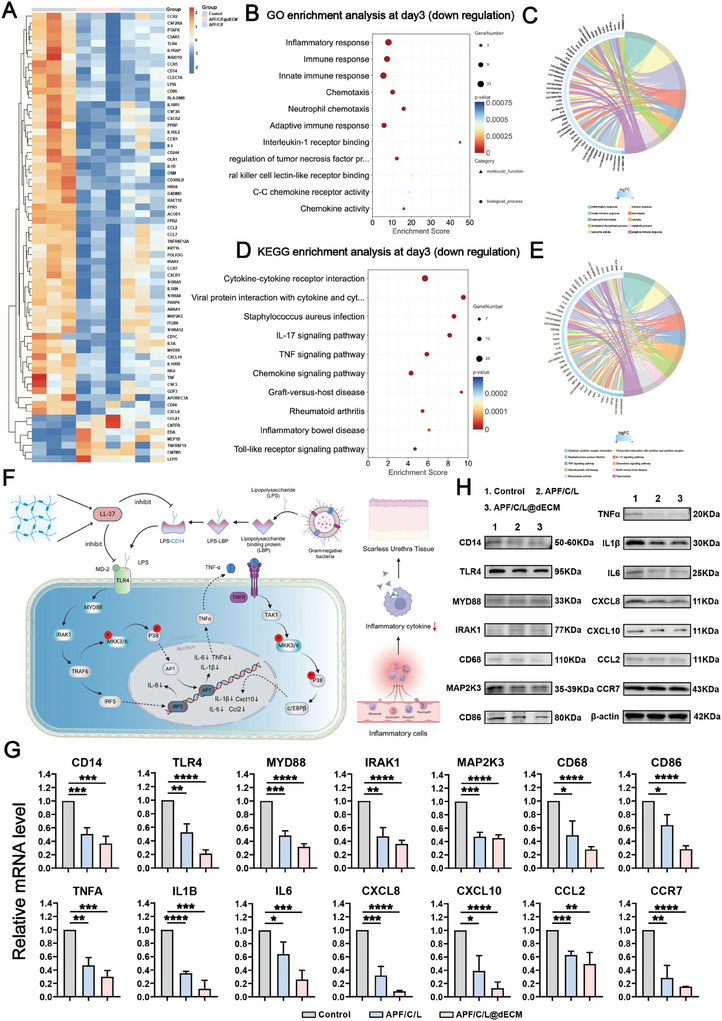
Schematic representation of the mechanism underlying the hydrogel‐mediated scarless urethral wound repair process at 3 days after treatment initiation. A) Heatmap of inflammatory gene expression in the different treatment groups. B,C) GO enrichment analysis of the top 30 downregulated genes involved in inflammation. D,E) KEGG enrichment analysis of the top 20 downregulated genes involved in inflammation. F) Schematic diagram of the signaling pathway through which APF/C/L@dECM hydrogel exerts its anti‐inflammatory effects to promote urethral wound healing and scarless repair. G) The relative mRNA levels of genes associated with inflammation identified by RNA sequencing in urethral wound tissue were assessed using real‐time qPCR (*n* = 3 independent biological samples per group). H) Representative WB generated using tissue samples taken from around the wound site at 3 days after injecting rabbits with saline (control), APF/C/L, or APF/C/L@dECM hydrogel. Data are expressed as the mean ± standard deviation (SD) (*n* = 3). *p < 0.05, **p < 0.01, ***p < 0.001, ****p < 0.0001.

Since the cells are more active in the primary stage of wound repair, to further explores the mechanisms underlying the ability of the hydrogel to promote urethral wound repair and scarless reconstruction, we performed RNA‐seq on the urethral tissue at 3 and 7 days after treatment initiation. We first analyzed the differentially expressed genes (DEGs) at 3 days after treatment initiation. We used kyoto encyclopedia of genes and genomes (KEGG) enrichment analysis to examine the top 20 downregulated genes associated with inflammation, and then performed a gene ontology (GO) enrichment analysis of the top 30 downregulated inflammatory genes (Figure S22A,B,E,F, Supporting Information). We identified several inflammation‐related genes among these sets (**Figure** **6**B–E). The clustering heatmap of DEGs involved in inflammation highlighted significant differences in gene expression among the different experimental groups (Figure 6A). For instance, the expression of inflammatory genes was significantly suppressed in the APF/C/L and APF/C/L@dECM groups, which further confirmed that the LL‐37 included in our hydrogel preparations had a substantial anti‐inflammatory effect. To delineate the immunomodulatory mechanisms of the hydrogels, we further investigated their effect on the cytokine/cytokine receptor interaction network, the TNF signaling pathway, and the Toll‐like receptor (TLR) signaling pathway (Figure S22C,D, Supporting Information).

The proposed mechanism by which the hydrogel preparations may mediate scarless urethral wound healing is illustrated in Figure 6F. Briefly, we speculated that LL‐37 inhibited the assembly of the lipopolysaccharide (LPS) receptor complex and bound to the CD14 and TLR‐4 receptors, preventing receptor‐mediated LPS capture. This inhibition event subsequently downregulated the expression of downstream signaling molecules, such as MYD88, IRAK1, and MKK3/6, which ultimately reduced the expression of multiple inflammatory cytokines (including IL‐1B, TNFA, and IL6), thereby exerting immunomodulatory effects and promoting scarless urethral repair. To validate this hypothesis, we analyzed the expression of the abovementioned genes and inflammatory cytokines across the different treatment groups (Figure S23, Supporting Information). PCR was then used to analyze the relative mRNA expression levels of inflammatory genes in the control, APF/C/L and APF/C/L@dECM groups (Figure 6G). Additionally, WB analysis confirmed that LL‐37 suppressed the expression of receptors, key signaling pathway components, and downstream inflammatory cytokines at the protein level, which likely represented a crucial aspect of the scarless wound healing process (Figure 6H).

**Figure 7:**
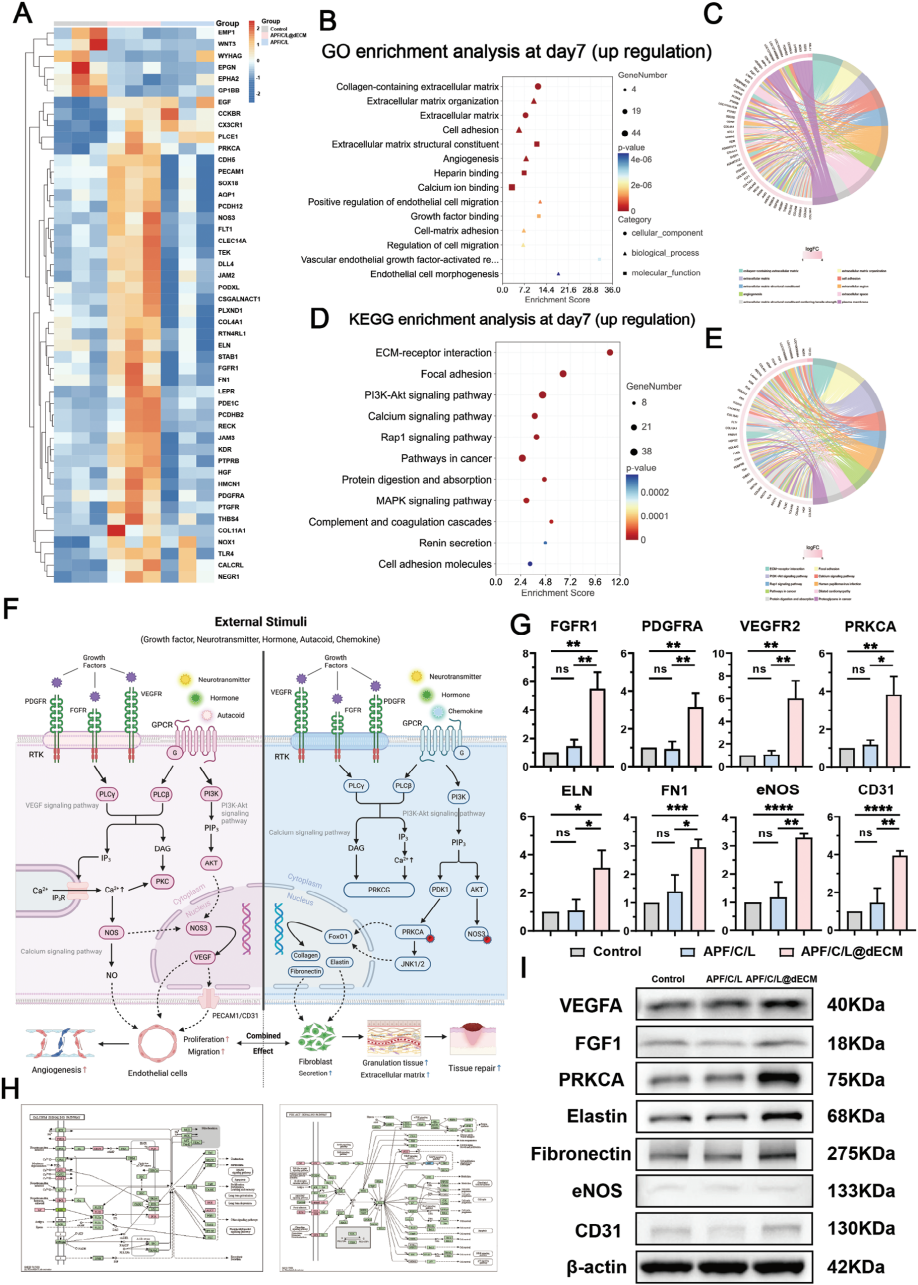
Schematic representation of the mechanism underlying the hydrogel with programmed regulation of wound healing performance at day 7 after treatment initiation. A) Heatmap of regenerative and angiogenic genes expressed in the different treatment groups. B,C) GO enrichment analysis of the top 30 upregulated genes involved in regeneration and vascularization. D,E) KEGG enrichment analysis of the top 20 upregulated genes involved in regeneration and vascularization. F) Schematic diagram of the signaling pathway through which APF/C/L@dECM hydrogel achieves scarless urethral wound healing. G) Relative mRNA levels of the genes associated with increased matrix secretion and vascularization identified by RNA sequencing in urethral wound tissue were assessed using real‐time qPCR (*n* = 3 independent biological samples per group). H) A comparison of the PI3K‐Akt and calcium signaling pathways between the control and APF/C/L@dECM groups. I) Representative images of WB generated using tissue samples taken from around the wound at 7 days after the injection of saline, APF/C/L, or APF/C/L@dECM into rabbits. Data are expressed as the mean ± standard deviation (SD) (*n* = 3). ns, not significant (*p* > 0.05), *p < 0.05, **p < 0.01, ***p < 0.001, ****p < 0.0001.

To investigate the role of the ADSCs dECM component of the programmed hydrogel in promoting cell proliferation and tissue regeneration during the later stages of wound healing, we repeated the above analyses at 7 days after treatment initiation (Figure S24A, Supporting Information). The analysis of upregulated GO‐enriched DEGs revealed that they were primarily implicated in angiogenesis and ECM regeneration. Meanwhile, KEGG enrichment analysis indicated that genes involved in ECM/receptor interactions, the PI3K‐Akt signaling pathway, and calcium signaling were significantly upregulated in the APF/C/L@dECM group (**Figure** **7**B–E; Figure S24B–F, Supporting Information). Notably, Gene Set Enrichment Analysis (GSEA) confirmed the increased expression of pathways related to angiogenesis and tissue regeneration, two processes that are critical for effective wound healing, in the APF/C/L@dECM group (Figure S25, Supporting Information). The differential gene clustering heatmap supported these results (Figure 7A), suggesting that the ADSC dECM component of the hydrogel promoted local angiogenesis, tissue regeneration, ECM reconstruction.

After conducting further analysis of the PI3K‐Akt and calcium signaling pathways, we were able to illustrate the underlying mechanisms (Figure 7F,H). We envisaged that the growth factors enriched in the dECM powder activated receptor tyrosine kinases (RTKs) in the calcium signaling pathway, promoting the expression of downstream phospholipase C β (PLCβ) and inositol triphosphate (IP3). The IP3 subsequently bound to and activated its receptor, IP3R, leading to the release of calcium ions from the endoplasmic reticulum, which promoted the expression of the nitric oxide synthase (NOS). Additionally, local neurotransmitters, hormones, and endocrine factors activated G‐protein‐coupled receptors, enhancing the expression of downstream protein kinase C alpha (PRKCA) and endothelial (e)NOS via the PI3K‐Akt signaling pathway. Collectively, these two signaling pathways synergistically promoted NOS expression, thereby stimulating the secretion of NO and VEGF, and activating the transmembrane glycoprotein CD31. These events ultimately facilitated the proliferation and migration of endothelial cells, promoting angiogenesis. Moreover, the upregulation of the PRKCA gene promoted the expression of regeneration‐related genes such as collagen, elastin, and fibronectin, thereby inducing fibroblasts to secrete ECM in favor of wound healing and tissue regeneration. PCR and WB confirmed the high expression of the key genes/proteins involved in the abovementioned signaling pathways, angiogenesis, and tissue regeneration in the APF/C/L@dECM group (Figure 7G,I)

### 2.6 Full‐Spectrum Validation of Whole‐Process Repair Hydrogel with Programmed Regulation of Wound Healing Performance

**Figure 8:**
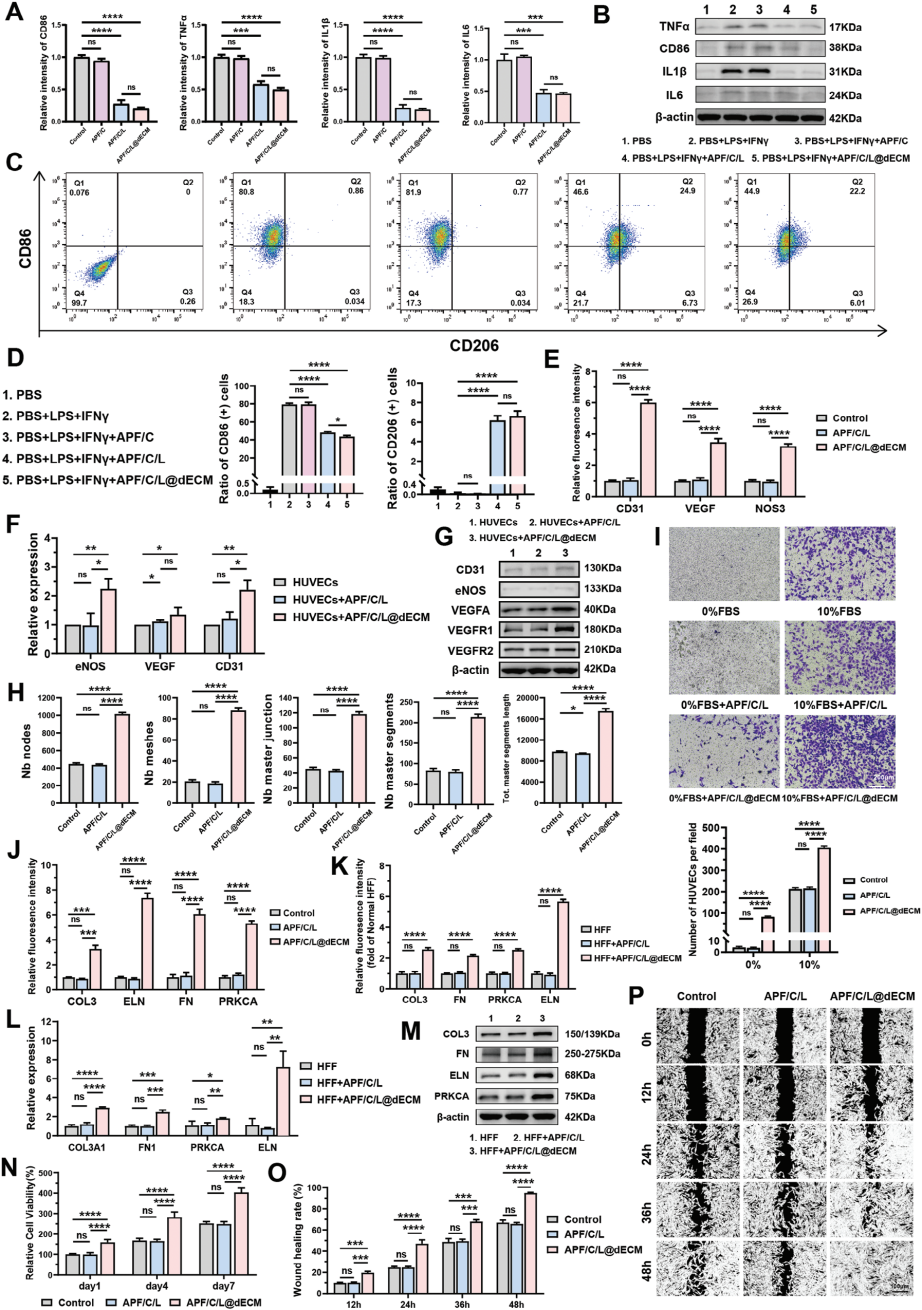
Multidimensional validation of hydrogel for wound healing in vivo and in vitro. A) Relative fluorescence intensity of CD86, TNFα, IL1β, and IL6 in immunofluorescence staining of rabbit urethra after different treatments (i.e., saline, APF/C, APF/C/L, and APF/C/L@dECM) for 3‐days. B) The protein expression levels (TNFα, CD86, IL1β, and IL6) of RAW264.7 cells in different hydrogels after 3‐days culture. C,D) The RAW264.7 cells were cultured with different treatments in the presence or absence of LPS and IFN‐γ for 24 h. The ratio of M1 and M2 macrophage was measured by FCM analysis. E) Relative fluorescence intensity of CD31, VEGF, and NOS3 in immunofluorescence staining of rabbit urethra after different treatments for 7‐days. The corresponding gene F) and protein G) expression levels (CD31, eNOS, VEGFA, and VEGFR) of HUVECs in different hydrogels after 7‐days culture. The tube formation and migration ability of HUVECs with different treatments were measured by in vitro angiogenesis assay H) and Transwell migration assay I). Relative fluorescence intensity of COL3, ELN, FN, and PRKCA in immunofluorescence staining of rabbit urethra J) and HFF K) after different treatments for 7‐days. The corresponding gene L) and protein M) expression levels (COL3, ELN, FN, and PRKCA) of HFF in different hydrogels after 7‐days culture. N) The cell proliferation rates of HFF at 1, 4, and 7 days after different treatment detected by the CCK‐8 assay. O,P) Dark‐field images (and corresponding quantitative data obtained) of HFF cultured under different conditions for 12, 24, 36, or 48 h in a wound healing assay. Data are expressed as the mean ± standard deviation (SD) (*n* = 3/6). ns, not significant (*p* > 0.05), *p < 0.05, **p < 0.01, ***p < 0.001, ****p < 0.0001.

To more comprehensively and further substantiate the capabilities of the whole‐process repair hydrogel, including its synergistic promotion of angiogenesis, matrix regeneration, and immunomodulation, we conducted extensive validations both in vivo and in vitro. At 3 days post‐treatment, the immunofluorescence analysis of urethral sections revealed a significant reduction in inflammatory indexes in both the APF/C/L and APF/C/L@dECM groups (**Figure** **8**A; Figure S26, Supporting Information). We next validated the anti‐inflammatory properties of the hydrogel in vitro by using RAW264.7 cells. The result of WB demonstrated that the expression of markers such as TNFα, CD86, IL‐6, and IL‐1β significantly decreased, after coculture with APF/C/L or APF/C/L@dECM (Figure 8B). Flow cytometry (FCM) results indicated a similar trend. We pretreated cells with different hydrogels for 12 h, followed by treatment with 100 ng mL^−1^ LPS and 20 ng mL^−1^ IFN‐γ for 24 h to induce macrophage polarization. The results showed that the proportion of CD86+ M1 macrophages significantly decreased in the APF/C/L and APF/C/L@dECM groups, while the proportion of CD206+ M2 macrophages slightly increased slightly (Figure 8C,D). These findings suggest that both APF/C/L and APF/C/L@dECM hydrogels exhibit excellent anti‐inflammatory effects in vitro and in vivo due to the presence of the LL‐37 antimicrobial peptide.

We next conducted additional analyses to validate the effects of the hydrogels on angiogenesis and ECM secretion. We began by verifying the angiogenic capability of the hydrogels by collecting urethral wound tissues from different treatment groups and subjecting them to immunofluorescence staining for eNOS, VEGF, and CD31. The expression of these markers significantly increased in the regenerated areas of the APF/C/L@dECM‐treated group, suggestive of new blood vessel formation (Figure 8E; Figure S27, Supporting Information). Both PCR and WB confirmed that coculturing human umbilical vein endothelial cells (HUVECs) with APF/C/L@dECM for 48 h increased their expression of genes/proteins implicated in vascularization (Figure 8F,G). Similarly, tube formation assays demonstrated that APF/C/L@dECM treatment promoted cell angiogenic effect (Figure 8H; Figure S28, Supporting Information). Subsequently, we evaluated the effect of APF/C/L@dECM hydrogel on the migration ability of HUVECs by Transwell migration assay. HUVECs were placed in the upper chamber with serum‐free medium, while the lower chamber contained either serum‐free medium or medium with 10% FBS, along with different hydrogels. The results indicated that the APF/C/L@dECM hydrogel significantly promoted cell migration (Figure 8I).

To further validate the ability of the hydrogel to promote local ECM secretion and tissue regeneration, we subjected urethral wound tissues harvested from rabbits at 7 days after treatment injection to immunofluorescence staining for COL3, FN1, PRKCA, and ELN. We found that the APF/C/L@dECM‐treated group expressed high levels of genes associated with ECM secretion and regeneration at the wound site (Figure 8J; Figure S29, Supporting Information). We also performed immunofluorescence staining experiments with human foreskin fibroblasts (HFFs), which confirmed that the APF/C/L@dECM substantially boosted the secretion of ECM from HFFs (Figure 8K; Figures S30 and S31, Supporting Information). These results were confirmed by findings from PCR and WB analyses (Figure 8L,M). Moreover, CCK‐8 and wound healing assays demonstrated that APF/C/L@dECM enhanced fibroblast proliferation and migration (Figure 8N–P; Figure S32, Supporting Information).

## 3 Discussion

Urethral injury is a common urologic disease. The harsh local microenvironment of the injured urethra dysregulates normal healing mechanisms, leading to scar tissue formation in the later stages of the process. Excessive scarring ultimately leads to urethral stricture, which considerably impacts patients' quality of life.^[^ ^21^ ^]^ There are many etiologies of urethral injuries, the main ones being urethral trauma associated with pelvic fracture or straddle injuries and iatrogenic injuries associated with urethral instrumentation (e.g., catheterization and cystoscopy), surgery for prostate, or radiotherapy.^[^ ^22^ ^]^ In clinical practice, the primary treatment principle for patients with urethral trauma is to restore urethral patency.^[^ ^23^ ^]^ For patients with mild injury and successful gentle catheterization attempts, most physicians prefer to use an indwelling catheter for 3 weeks.^[^ ^24^ ^]^ However, in cases where urethral damage is severe, such as urethral rupture leading to failed catheterization, emergency physicians often choose suprapubic cystostomy or early endoscopic realignment.^[^ ^25^ ^]^ Early endoscopic realignment can quickly restore urethral continuity with minimal injury, shorter operative time, and faster recovery.^[^ ^26^ ^]^ Nevertheless, due to the disruption of urethral tissue function and structure during the acute injury phase and the poor healing microenvironment, the incidence of urethral stricture post‐procedure is relatively high. Consequently, more emergency physicians are opting for suprapubic cystostomy to maintain urethral patency and allow the “Urethral Rest” period.^[^ ^27^ ^]^ This approach provides time for urethral tissue recovery and stabilization of scar tissue before undergoing urethral reconstruction. However, second‐stage surgery can prolong recovery time, increase the risk of complications, and impose psychological and financial burdens on patients. For iatrogenic injuries, indwelling catheterization for temporary support of the urethra is usually preferred without specific treatment.^[^ ^28^ ^]^ Overall, regardless of the emergency management strategy, current clinical treatments focus solely on restoring urethral continuity and patency after injury, without addressing urethral wound healing and the prevention or minimization of subsequent scar formation.^[^ ^29^ ^]^ More importantly, many of the treatments available in clinical practice today, such as urethroplasty, are aimed at situations in which a urethral stricture has already occurred following a urethral injury.^[^ ^4^, ^30^ ^]^ In response to the above problems, we innovatively designed a whole‐process repair hydrogel with programmed regulation of wound healing performance, aiming at early endoscopic realignment along with localized in situ injection of hydrogel at the injury site for synergistic treatment. This approach not only promotes early urethral wound healing, but also prevents or minimize scar tissue formation and reduces the burden on patients. The hydrogel has a high potential for clinical application and may offer a new perspective for more efficient and convenient treatment of urethral injury in clinical practice.

Briefly, in this study, our group developed a novel hydrogel that could be injected in situ and can carry a number of components with essential roles at each stage of urethral wound repair. For instance, in the hemostatic phase, collagen binds to certain sites on the surface of localized platelets or blood cells in the wound, increasing their aggregation and deformation to promote blood coagulation.^[^ ^31^ ^]^ In the inflammatory stage, the accumulation of urine and necrotic tissue in the urethral wound can favor bacterial growth. In such a setting, the LL‐37 antimicrobial peptide not only exerts a powerful antibacterial effect but also inhibits local inflammatory reactions to remodel the microenvironment in favor of wound healing. In the proliferation phase, ADSCs sheet dECM, which are rich in various growth factors and nutrients, promote ECM regeneration and angiogenesis, the proliferation and migration of fibroblasts and vascular endothelial cells, as well as their ability to secrete key soluble factors. This restores blood flow to the wound site and increases the deposition of ECM at the would site to promote healing. Endowing our hydrogel (APF/C/L@dECM) with all the above capabilities ensured that it could be injected locally into the urethral wound to reduce inflammation and promote angiogenesis and tissue regeneration. Our experiments in rabbits confirmed that APF/C/L@dECM also altered the collagen composition and regulated cell proliferation at the wound site, which ultimately prevented the formation of scar tissue.

In recent years, many academics have found that Urethral injury disrupts the balance of collagen synthesis and degradation in the local ECM, which alters collagen composition and triggers a variety of cellular changes, which result in ECM remodeling and scar tissue formation.^[^ ^32^ ^]^ In addition, these cellular changes activate myofibroblasts.^[^ ^33^ ^]^ Two mechanisms of myofibroblast activation have been identified to date. Myofibroblasts can either be activated by chemical factors (e.g., TGF‐β and its downstream classical Smad 2/3 pathway and the non‐classical JNK pathway) or by physical factors (e.g., mechanical stimulation).^[^ ^34^ ^]^ The primary focus of the present study was to reprogram urethral tissue regeneration and ECM deposition to promote would healing while limiting late‐stage scar tissue formation. There are limitations, however, in this repair system for the regulation of myofibroblasts activation as well as myofibroblasts apoptosis and clearance. Considering the evidence supporting the potential benefits of inhibiting myofibroblasts overactivation (and thereby adverse ECM remodeling) promoting later‐stage myofibroblasts apoptosis on scarless urethral wound repair, further studies exploring the role of these cells in the context of urethral wound healing may help the development of more effective treatment strategies.

## 4 Conclusion

Overall, we propose an integrated whole‐process repair system (APF/C/L@dECM), delivered by an in situ injectable, thermoreversible hydrogel. Current clinical treatments for urethral strictures primarily focus on urethras with existing scar tissue, while little attention is given to the healing of the urethral wound post‐injury and the prevention or reduction of scar tissue formation. The APF/C/L@dECM repair system designed in this study can be effective in the hemostasis, inflammation, and proliferation phases of urethral injury, thereby promoting wound healing and preventing scar tissue formation. We have validated the efficacy of the repair system in preventing scar formation following urethral injury in rabbits. Additionally, we elucidated the mechanisms by which APF/C/L@dECM exerts anti‐inflammatory effects during the inflammatory phase and promotes angiogenesis and extracellular matrix secretion during the proliferative phase of injury repair, but there are some deficiencies in the regulation of myofibroblasts. Our findings indicate that the APF/C/L@dECM hydrogel is simple to prepare and easy to use, with significant potential for clinical application, offering new insights for the treatment of urethral injuries in a clinical setting.

## 5 Experimental Section

### Synthesis of Amino‐Terminated PF127 Copolymer (APF)

Dissolve 20 g of PF127 (Sigma–Aldrich, P2443) in 300 mL of dichloromethane (Sigma–Aldrich, 270997), add 15 mL of triethylamine (Sigma–Aldrich, 2471283), and stir in an ice‐water bath until well mixed. Use a syringe to inject 7.5 mL of methanesulfonyl chloride (MsCl) (Sigma‐Aldrich, 8.06021) under the liquid surface in two batches to avoid excessive exotherm and white smoke. The reaction was carried out at room temperature for 12 h. The reaction solution was filtered to remove solids, rotary distilled to almost no sol, and poured into petroleum ether to obtain a solid. After drying, the solid was dissolved in 200 mL of ammonia and stirred vigorously for 3 days. After standing until the foam disappeared, the reaction was extracted with dichloromethane three times, and the dichloromethane phase was dewatered with anhydrous magnesium sulfate to obtain the APF.

### Synthesis of LL‐37 Antimicrobial Peptide

Briefly, take appropriate amount of modified resin for peptide synthesis, first add 20% pip/DMF to the reactor and shake the reaction for 20 min. Filter to remove the solvent, add DMF to the system, shake the reactor for 1 min and filter to remove the liquid; this operation was performed three times. Take appropriate amount of detection reagents A and B and a little resin and add them to the detection tube, put the tube into 100 °C for 30–60 s, check whether there was any color change of the resin, if color changes, it means that the Fmoc removal was successful. The configured amino acid solution was added to the reactor, DIC solution was added and the reactor was shaken for 1 h. Subsequently, the condensation reagent was added for cyclization modification. The resin was cleaved and analyzed by crude assay and finally purified by RP‐HPLC and fractions were collected and lyophilized. The LL‐37 antimicrobial peptide (LLGDFFRKSKEKIGKEFKRIVQRIKDFLRNLVPRTES) was synthesized with the help of GenScript Biotech Corporation (Nanjing, China) with purities greater than or equal to 98%.

### Synthesis of APF/C‐L@dECM Hydrogel

10 g/20 g/30 g of APF solids were dissolved in 100 mL of deionized water and stirred overnight at 4 °C to obtain 10%, 20%, and 30% wt. APF solutions. Subsequently, 5 g/10 g/20 g of fish collagen peptide powder (Aladdin, F573426) and 2% wt. EDC (Aladdin, E106172) and 2% wt. NHS (Sigma–Aldrich, 130672) powder was added to the 20% wt. APF solution (APF20) for crosslinking and stirred overnight at 4 °C to obtain 5% wt. collagen APF20 (APF20/C5) hydrogel, 10% wt. Collagen APF20 (APF20/C10) hydrogel and 20% wt. collagen APF20 (APF20/C20) hydrogel. Finally, 0.02% wt./0.1% wt./0.5% wt. LL‐37 antimicrobial peptide powder was added to APF20/C10 hydrogel to obtain APF20/C10/L‐0.02, APF20/C10/L‐0.1 and APF20/C10/L‐0.5 hydrogels. The specific concentrations and the weight ratios of each component are shown in **Table** **1**.

**Table 1:** Concentrations of each component in different hydrogels. The text in brackets of the leftmost column corresponds to the abbreviation of the long name, which is used in the content of the article.

Hydrogel	APF [wt.%]	Collagen [wt.%]	LL‐37 [wt.%]	dECM [mg mL^−1^]	EDC/NHS [wt.%]
APF10	10	/	/	/	/
APF20	20	/	/	/	/
APF30	30	/	/	/	/
APF20/C5	20	5	/	/	2
APF20/C10 (**APF/C**)	20	10	/	/	2
APF20/C20	20	20	/	/	2
APF20/C10‐L‐0.02	20	10	0.02	/	2
APF20/C10‐L‐0.1	20	10	0.1	/	2
APF20/C10‐L‐0.5(**APF/C/L**)	20	10	0.5	/	2
APF20/C10‐L‐0.5@dECM5	20	10	0.5	5	2
APF20/C10‐L‐0.5@dECM10	20	10	0.5	10	2
APF20/C10‐L‐0.5@dECM20	20	10	0.5	20	2
APF20/C10‐L‐0.5@dECM40	20	10	0.5	40	2
APF20/C10‐L‐0.5@dECM80(**APF/C/L@dECM**)	20	10	0.5	80	2
APF20/C10‐L‐0.5@dECM160	20	10	0.5	160	2

### Harvest of rADSCs Sheet dECM Powder

Briefly, fresh adipose tissue was washed three times with phosphate‐buffered saline (PBS) and then cut into small pieces with sterile scissors. The tissue pieces were then digested with collagenase (Nordmark, S1745401) solution at a concentration of 1 mg mL^−1^ for 2 h to obtain a cell suspension. The cell suspension was filtered through a 200 µm special sterile filter and centrifuged. Primary ADSCs were collected and cultured in DMEM (Dakewe, 66016111) supplemented with 10% FBS (YUKA Biotech, Shanghai, China, YK‐1001‐5010) and 1% penicillin/streptomycin (NEST Biotechnology Co. Ltd., 211092). When cultured to the P2 generation, 20 mg mL^−1^ vitamin C (Sigma–Aldrich, PHR1008) was added to the medium and the serum concentration was increased to culture the cells into cell membrane sheets. The obtained cell membrane sheets were immersed in decellularization solution A and shaken at a frequency of 100–150 r min^−1^ for 48 h at 37 °C on a shaker. Decellularization solution A was composed of PBS buffer containing 10 mm Tris‐HCl (Sigma–Aldrich, 10812846001), 10 mm EDTA (Sigma–Aldrich, 324 504), 1% TritonX‐100 (Solarbio, T8200) and 1% SDS (Solarbio, S1010). The tissues were then placed in decellularization solution B and shaken at 100–150 r min^−1^ for 24 h. Decellularization solution B consists of PBS buffer containing 400 µg mL^−1^ of deoxyribonuclease I (Solarbio, D8071). The decellularized samples were lyophilized and incubated with PBS buffer. The decellularized samples were lyophilized and ground to a powder using a grinder and then sterilized by C060γ irradiation.

### Absorption and Degradation Ratio Assay In Vitro

The water absorption capacities of various hydrogels, which was also called as SR were measured. The different samples (APF20, APF20/C10 and APF20/C10/L‐0.5@dECM) were re‐immersed in PBS buffer at 35 °C for a certain time. After PBS were removed at certain time points, the hydrogels were gently scrubbed using absorbent paper to remove the residual solvent, and weights at each time points were measured. The SR values of hydrogel were calculated according to the Equation:

(1) $$\mathrm{SR}=\frac{\left(Wo-Wt\right)}{Wo}\times 100\%$$

Note: wt. is the weight of hydrogel after 0.5, 1, 2, 10, 30, 60, 120, 240, and 360 min of absorption, and W_0_ is the initial weight of hydrogel.

The degradation characteristics of APF20, APF20/C10, and APF20/C10/L‐0.5@dECM hydrogels were measured. At the beginning of the experiment, three groups of hydrogels with the same quality were selected, with three samples in each group. The hydrogels were placed in 5 mL of PBS buffer and 5 mL of urine respectively and degraded in a shaker at 35 °C and 100 rpm. At the end of shaking, the samples were collected and lyophilized, and then their weights were measured separately. The degradation rate was calculated according to the Equation:

(2) $$D=\frac{\left(\mathrm{Wo}-\mathrm{Wt}\right)}{\mathrm{Wo}}\times 100\%$$

Note: wt. is the mass of residual hydrogel after 1, 3, 5, 7, 9, 11, and 13 days of degradation, and W_0_ is the initial mass of hydrogel.

### Rheology Analysis of Hydrogel

The rheological properties of hydrogels were tested by monitoring the in‐situ gelation process using a temperature scanning method (HAAKE RheoStress 6000, Thermo Scientific, USA). The tests were performed using a 10 mm diameter fixture. The temperature was first cooled down to 5 °C and subsequently increased at a rate of 1 °C min^−1^. The constant strain was 1%, the frequency 0.1 rad s^−1^, and the temperature range was 5–50 °C. The storage modulus (G′) and loss modulus (G″) were recorded as a function of temperature.

### Amino Acids Analysis

The substances (15 mg) were accurately weighed and hydrolyzed in 10 mL HCl (6 m) at 110 °C for 22–24 h. The solution was diluted to 25.00 and 1.00 mL solution was evaporated in a water bath at 60 °C. The samples were redissolved in sample diluent and the amino acid content was determined using an amino acid analyzer (A300, Membra Pure GmbH, Germany). The assay results were output by the chromatographic data processing system. The data processing system exported the assay results.

### X‐Ray Photoelectron Spectroscopy (XPS)

XPS measurements of PF127, APF, APF/C, APF/C‐L, and APF/C‐L@dECM hydrogels were performed for peptide conjugation assessment using an ESCALAB QXi XPS spectrometer (Thermo Fisher Scientific, USA). For analysis, hydrogels were lyophilized, and powdered compacts were laid flat on conductive carbon tape. The samples were analyzed using micro–focused radiation with a spot size of 400 µm, and fine spectral tests were performed on the C and N orbitals.

### Hemolysis Experiment

Fresh anticoagulated blood was drawn from the ear marginal vein of healthy New Zealand rabbits. 10 mL of distilled water, 10 mL of saline, 10 mL of saline+1 mL of APF, 10 mL of saline+1 mL of APF/C5, 10 mL of saline+1 mL of APF/C10 were added to each of the five sets of tubes, which were then mixed thoroughly and incubated for 60 min at 37 °C. The tubes were removed and centrifuged at 2500 rpm for 5 min. The liquid in each tube was removed and centrifuged at 2500 rpm for 5 min. The supernatant was then taken and the absorbance was measured with a UV spectrophotometer at a wavelength of 545 nm, repeated three times and averaged. The hemolysis rate was defined as (sample absorbance‐negative control absorbance) / (positive control absorbance‐negative control absorbance) *100% and repeated three times.

### BCI Experiment

0.2 mL of APF, APF20/C5, and APF20/C10 hydrogels were placed into clean centrifuge tubes and then the centrifuge tubes containing the materials were transferred into a water bath and left to stand for 5 min at 37 °C. Then, 0.1 mL of anticoagulated whole blood was dropped on the material and 200 uL of 0.2 m calcium chloride solution (Sigma–Aldrich, 21115) was added immediately. After 5 min, 25 mL of deionized water was added to each centrifuge tube and transferred it to an oscillating water bath in a pot at 37 °C with a water flow rate of 50 rpm for 5 min. After that, the solution was removed from the centrifuge tubes and measured the absorbance value (A1) of the solution in each centrifuge tube using a UV–vis spectrophotometer at 545 nm. 0.1 mL of blood was taken, 25 mL of deionized water was added, and the absorbance value (A2) was measured at the same wavelength as the reference value. This procedure was repeated three times and the coagulation index BCI was calculated using the formula BCI = A1/A2.

### Dynamic Clotting Time

Six portions each of 0.2 mL of APF, APF20/C5, and APF20/C10 hydrogel were taken into clean centrifuge tubes. Seven‐time points were set up with a time interval of 5 min between each two adjacent points. Then, 200 uL anticoagulated whole blood was taken on the surface of each material. The same procedure was repeated for each material in each petri dish. Once the procedure was completed, a timer was started, and added 50 mL of distilled water to the centrifuge tube every 5 min. After 5 min of standing, the leachate was recovered and the optical density of free hemoglobin in the leachate was measured at different time points using a spectrophotometer at a wavelength of 540 nm, and then a dynamic coagulation curve was plotted.

### Dynamic Clotting Time

The number of platelets adhering to the surface of the hydrogel was measured using the LDH assay kit (Beyotime, China). Platelet‐rich plasma (PRP) was obtained by centrifuging anticoagulated whole blood (1000 g) for 12 min. Diluted platelets were lysed with 1% TritonX‐100, and the released LDH was quantified at 450 nm absorbance using an LDH detection kit, and a standard curve of platelet count versus absorbance was plotted. Different groups of hydrogels were placed on 24‐well plates, and then 100 µL of PRP was added to their surface and stored and incubated at 37 °C. At 30 and 60 min, unadhered platelets were washed off from each group of hydrogels with PBS, and then detected by using the LDH assay kit, and the standard curves were compared.

### In Vitro Antibacterial Assay

Paper sheets moistened with different groups of solutions were placed on solid nutrient medium uniformly coated with bacterial suspensions and incubated at 37 °C for 12–24 h before the final measurement of the size of the ring of inhibition.

Bacterial suspensions of a certain bacterial concentration (≈10^6^ CFU mL^−1^) were incubated with different gels for 12 h. At the end of the incubation, the bacterial suspensions were inoculated onto the surface of solid nutrient medium and incubated at 37 °C for 12–24 h. After that, the petri dishes were taken out and photographed for evaluation.

After the plate was coated with bacteria, the live and dead bacteria were stained with green and red color using the LIVE/DEADTM BacLightTM Bacterial Viability Kit (Thermo Fisher Scientific, L7012), respectively, and then microscopic observation of bacterial live and dead was carried out to semi‐quantitatively assess the percentage of bacterial death.

Bacteria after the action of different hydrogels were fixed by 2.5% glutaraldehyde solution at 4 °C for 40 min. Subsequently, after dehydration using an aqueous solution of ethanol, they were dried and subjected to SEM observation.

### Evaluation of Decellularization

Samples of harvested rADSCs sheets and rADSCs dECM were fixed with 4% paraformaldehyde, embedded in paraffin, and cut into 7‐µm‐thick sections, which were stained with HE, DAPI, Alcian blue, and Sirius Red to characterize changes in the components of rADSCs sheets before and after decellularization. sGAG was quantified using a Blyscan sGAG Assay Kit (Biocolor, Carrickfergus, UK). Collagen contents were quantified using a SircolTM Collagen Assay Kit (Biocolor). The DNA content of rADSCs sheets before and after decellularization was determined using a PicoGreen DNA kit (Invitrogen, USA). The concentrations of VEGF (U96‐1640E), PDGF‐BB (U96‐2901E), EGF (U96‐ 2054E), bFGF (U96‐1738E), and HGF (U96‐2401E) in the lysates of the rADSCs sheet extracts before and after decellularization were determined by using the enzyme‐linked immunosorbent assay (ELISA) kits (YOBIBIO, Shanghai, China) according to the instructions of the manufacturer.

### Biocompatibility

Cell Counting Kit‐8 (CCK‐8) (ABclonal, RM02823) was used to evaluate the biosafety of the hydrogels and the ability of rADSCs dECM to promote cell proliferation growth. Briefly, ADSCs were co‐cultured with hydrogels for 1, 4, and 7 days. At each time point, the hydrogels were removed and incubated with 100 µL of fresh culture medium containing 10 µL of CCK‐8 at 37 °C for 3 h. The absorbance of the supernatant at 450 nm was analyzed using a microplate reader and the optical density values were recorded.

Cell survival of ADSCs after 7 days of co‐culture with different hydrogels was assessed in vitro using a live/dead cell staining kit. Culture dishes (CellPro Biotechnology, 803100B) were rinsed three times with PBS and then treated with propidium iodide (PI) and calcein (Calcein‐AM) in FBS‐free medium at room temperature, followed by incubation in the dark for 15 min. Afterward, they were rinsed three times with PBS and photographed using a fluorescence microscope. The percentage of live and dead cells was calculated after counting with ImageJ software.

### In Vivo Assessment of Scarless Healing of Urethral Wounds in Rabbits

Forty male rabbits (6–8 months old/2.5–4 kg body weight) were divided into five groups: control, APF, APF/C, APF/C/L, and APF/C/L@dECM. Ventral wound healing was easily disturbed after multilayered suture, whereas the dorsal side was close to the corpus cavernosum without excess tissue damage, which was suitable for observation and evaluation, so the dorsal injury model was used in this study. The injury site was constructed 1 cm from the external urethral opening, which helped in localizing the tissue collection. Saline, APF, APF/C, APF/C/L, and APF/C/L@dECM hydrogels were injected around the wound and the rabbits were executed at 4 and 8 weeks respectively. Tissues from the injured sites were immersed and fixed in 4% paraformaldehyde solution for 24 h. After dehydration using graded ethanol, these tissues were embedded in paraffin and sections (4 µm) were examined histologically by HE staining and Masson staining.

### Immunofluorescence Analysis

The expression of CD31, PCNA, α‐SMA, COL1, and AE1/AE3 in the urethra of each group was analyzed by immunofluorescence staining. Primary antibody incubation: the blocking solution (3% BSA) was slightly removed and the slides were incubated with primary antibody overnight at 4 °C and then placed in a wet box with a small amount of water. Secondary antibody incubation: slides were washed three times with PBS (pH 7.4) in a shaker setup for 5 min each time. The target tissue was then covered with secondary antibody (properly conjugated with primary antibody) and incubated for 50 min at room temperature in the dark. DAPI staining: Slides were washed three times with PBS (pH 7.4) in a shaker setup for 5 min each time and then incubated with DAPI solution for 10 min at room temperature in the dark. Then the slides were incubated with DAPI solution for 10 min at room temperature in the dark, and the images were captured by a fluorescence microscope, the excitation wavelength of DAPI was 330–380 nm, the emission wavelength was 420 nm, and the color was blue; the excitation wavelength of FITC was 465–495 nm, and the color was green with the emission wavelength 515–555 nm; the excitation wavelength of CY3 was 510–560 nm, and the emission wavelength was 590 nm, and the color was red. Semi‐quantitative analysis was performed using ImageJ software. Antibody sources and Catalog Number are detailed in Table S1 (Supporting Information).

### Urethrography Analysis

At 8 weeks postoperatively, rabbits receiving different treatments underwent urethrography before sacrifice. An 8F catheter was inserted into the urethra and radiographs were taken after injection of iodine contrast into the urethra. The degree of urethral stricture was assessed by comparing the images of each group in terms of the ratio of the width of the urethral stricture to the total width of the urethra.

### Maximum Flow Rate (Qmax)

Rabbits were anesthetized 8 weeks after surgery, 150 mL of saline was injected into the bladder, and then the rabbits were awakened and waited to urinate. During this procedure, the maximum flow rate through the urine was measured using a flow rate detector.

### qRT‐PCR Analysis

Gene expression levels of cells extracted from new tissues on days 3 and 7 after different treatments were analyzed by qRT‐PCR. qRT‐PCR was performed using SYBR Green Pro Taq HS qPCR Kit II (Rox Plus) (ACCURATE BIOTECHNOLOGY (HUNAN) CO., LTD, Changsha, China (AG11719)) on a detection system. The forward and reverse primer sequences for GAPDH, CD14, TLR4, MYD88, IRAK1, MAP2K3, CD68, CD86, TNFA, IL1B, IL6, CXCL8, CXCL10, CCL2, CCR7, FGFR1, PDGFRA, VRGFR2, PRKCA, ELN, COL3A1, FN1, eNOS, VEGF, and CD31 are listed in Table S2 (Supporting Information). The expression levels of mRNA were quantified with β‐actin as an internal control and the data were determined based on the cycle threshold method as R = 2–ΔΔCT (*n* = 3).

### Western Blotting Analysis

Tissues were harvested on days 3 and 7 after different treatments and added to RIPA lysis buffer containing protease and phosphatase inhibitors. After lysis on ice for 3 h, the supernatant was collected by centrifugation. Each lane was loaded with a total of 25 µg of protein and electrophoresed using an 8–12% SDS‐PAGE gel. The target proteins on the SDS‐PAGE gel were transferred to a polyvinylidene difluoride membrane (PVDF; 0.45 µm) and then blocked with 5% blocking buffer for 1 h at room temperature. Primary antibodies against β‐actin, CD14, TLR4, MYD88, IRAK1, MAP2K3, CD68, CD86, TNFα, IL1β, IL6, CXCL8, CXCL10, CCL2, CCR7, PRKCA, ELN, FN, VEGFA, eNOS, CD31, and FGF1 were treated with PVDF membranes respectively at PVDF membranes were treated overnight at 4 °C. The membranes were then rinsed three times with TBST and incubated with the corresponding secondary antibodies for 1 h. The membranes were then visualized with a developer. Antibody sources and part numbers are detailed in Table S1 (Supporting Information).

### Detection of Macrophage Polarization

The cells were pre‐treated by additionally adding different hydrogels to the RAW264.7‐specific medium (Pricella Life Science & Technology Co., Ltd, CM‐0190) according to different groups for 12 h. Subsequently, in order to induce the polarization of RAW264.7 cells toward the M1 phenotype, LPS (100 ng mL^−1^) (Sigma–Aldrich, L2880) and IFN‐γ (20 ng mL^−1^) (GenScript, Z02916) were added to the original system and cultured them for 24 h. The ratio of M1 and M2 phenotypes was detected by FCM assay.

### FCM Examination

Add the PE‐anti‐CD86 antibody and RAW264.7 cells respectively, mix thoroughly and then incubate at room temperature for 10–30 min, avoiding light. Add 1 mL of flow staining buffer and centrifuge at 300 g for 5 min. After removing the supernatant, add 500 uL of 4% paraformaldehyde and fix it at room temperature for 20 min. After centrifugation to wash away the fixative, add 500 uL of saponin and permeabilized for 10 min. After centrifugation to remove the permeabilized solution, add the FITC‐anti‐CD206 antibody, mix well and incubate at room temperature for 30 min. Add 1 mL of flow staining buffer and centrifuge at 300 g for 5 min. Discard the supernatant and add 1 mL of flow staining buffer, and then test on the machine.

### Tube‐Formation

First, Matrigel (Yeasen Biotechnology (Shanghai) Co., Ltd., 40186ES08) was added to a 96‐well plate and incubated at 37 °C for 1 h. Subsequently, HUVECs were placed into wells coated with Matrigel and divided into groups according to the treatment received. After 6 h of incubation at 37 °C in an incubator with 5% CO2 concentration, three random visual zones were collected and analyzed for tube length and total branch points using ImageJ software. ImageJ software was used for the analysis.

### Transwell Migration Assay

Suspensions of HUVECs were seeded into the upper chambers of 24‐well trans‐well inserts containing serum‐free medium. The lower chambers were serum‐free DMEM medium (LONSERA, Shanghai Shuangru Biology Science & Technology Co., Ltd., ME105‐001), serum‐free medium containing APF/C/L hydrogel and serum‐free medium containing APF/C/L@dECM hydrogel to assess cell migration. After 48 h of incubation, cells on the bottom surface were stained with crystal violet staining solution (Beijing Solarbio Science & Technology Co., Ltd., G1059) and quantified under a light microscope.

### Ethics Declaration

All animals were purchased from the Laboratory Animal Center of Shanghai Sixth People's Hospital, and all animal experiments were approved by the Animal Welfare Ethics Committee of Shanghai Sixth People's Hospital under the approval number (2024‐0658).

### Statistical Analysis

Data were obtained from at least three independent experiments and all data were expressed as mean ± standard deviation (SD) (*n* = 3–6). Multiple comparisons were performed using one‐way ANOVA and Tukey's post hoc test. *, **, ***, ****represent *p* < 0.05, 0.01, 0.001, and 0.0001, respectively, *p < 0.05 was statistically significant. All statistical analyses were performed using SPSS 13.0 software (SPSS Inc.)

## Conflict of Interest

The authors declare no conflict of interest.

## Author Contributions

W.F., Y.W., K.Z., and M.Y. contributed equally to this work. Q.F., R.Y., and W.F. conceptualized the study. W.F., Y.W., K.Z., and M.Y. developed the methodology. W.J., M.L., X.X., and Y.W. conducted the investigation. W.F., Z.Y., and X.X. were responsible for visualization. Q.F., R.Y., and M.Y. supervised the study. W.F. and R.Y. wrote the original draft. Q.F. and R.Y. reviewed and edited the manuscript.

## Supporting information



Supporting Information

## Data Availability

The data that support the findings of this study are available from the corresponding author upon reasonable request.
