# Supplementary material for: An Integrated Whole‐Process Repair System with Programmed Regulation of Healing Performance Facilitates Urethral Wound Restoration and Scarless Reconstruction
Source: Adv Sci (Weinh). 2024 Dec 18;12(6):2409930. doi: 10.1002/advs.202409930 (PMC11809434; doi:10.1002/advs.202409930)
Supplement: Supplementary file 1 — Supporting Information [file ADVS-12-2409930-s001.docx]

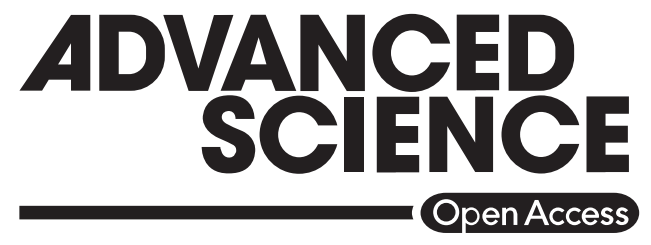


Supporting Information

for *Adv. Sci.,* DOI:

An Integrated Whole-Process Repair System with Programmed Regulation of Healing Performance Facilitates Urethral Wound Restoration and Scarless Reconstruction

*Wenzhuo Fang, Ying Wang, Kaile Zhang, Ming Yang, Meng Liu, Yangwang Jin, Xianjie Xiu, Yuhui Wang, Zhenwei Yu, Ranxing Yang*, Qiang Fu**


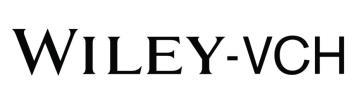


Supporting Information

**An Integrated Whole-Process Repair System with Programmed Regulation of Healing Performance Facilitates Urethral Wound Restoration and Scarless Reconstruction**

*Wenzhuo Fang, Ying Wang, Kaile Zhang, Ming Yang, Meng Liu, Yangwang Jin, Xianjie Xiu, Yuhui Wang, Zhenwei Yu, Ranxing Yang*, Qiang Fu**


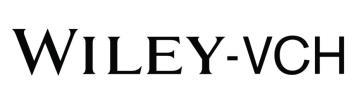


1. **Supplementary figures**


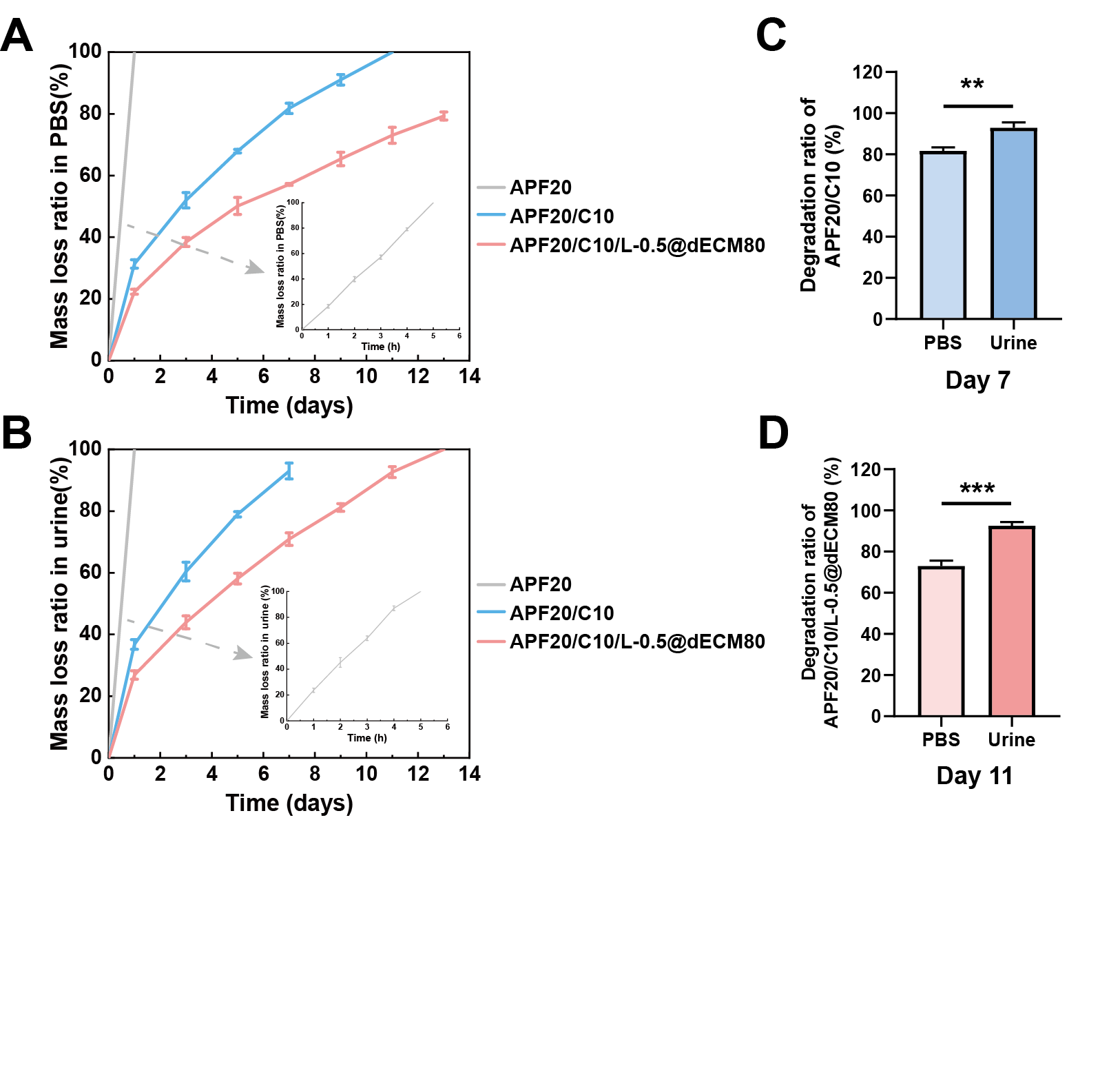


**Figure S1.** Degradation rates of above three hydrogels in PBS A) and urine B) at 35℃ as a function of time and their detailed values in PBS and urine after 7 days C) and 11 days D) incubation. Data are expressed as mean ± standard deviation (SD) (n = 3). **P < 0.01, ***P < 0.001.


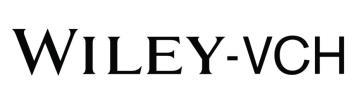


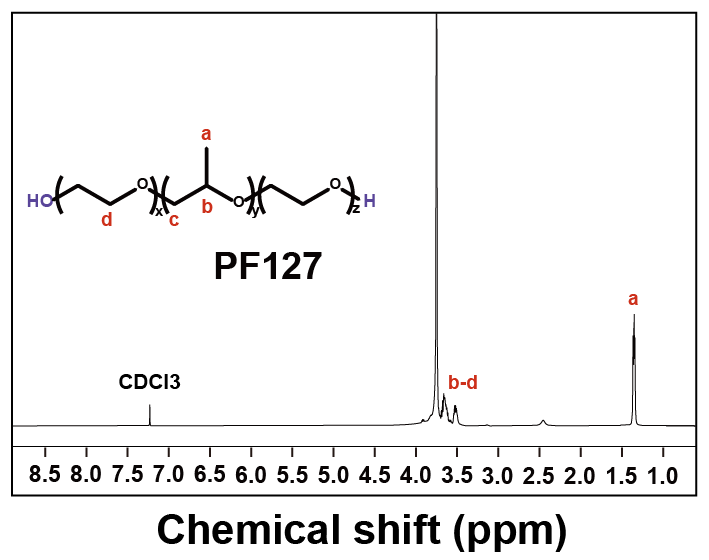


**Figure S2.** ^1^H NMR spectra of the PF127 in CDCl_3_.


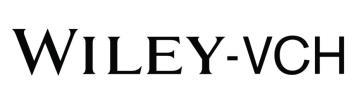


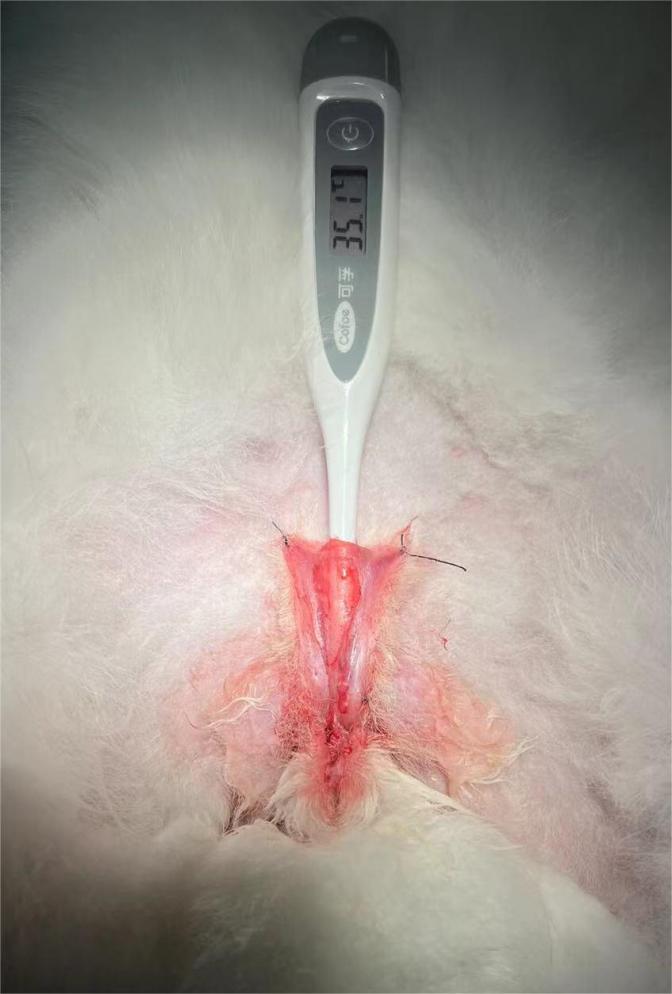


**Figure S3.** Schematic diagram of normal rabbit urethral temperature measured by thermometer.


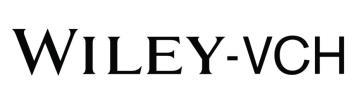


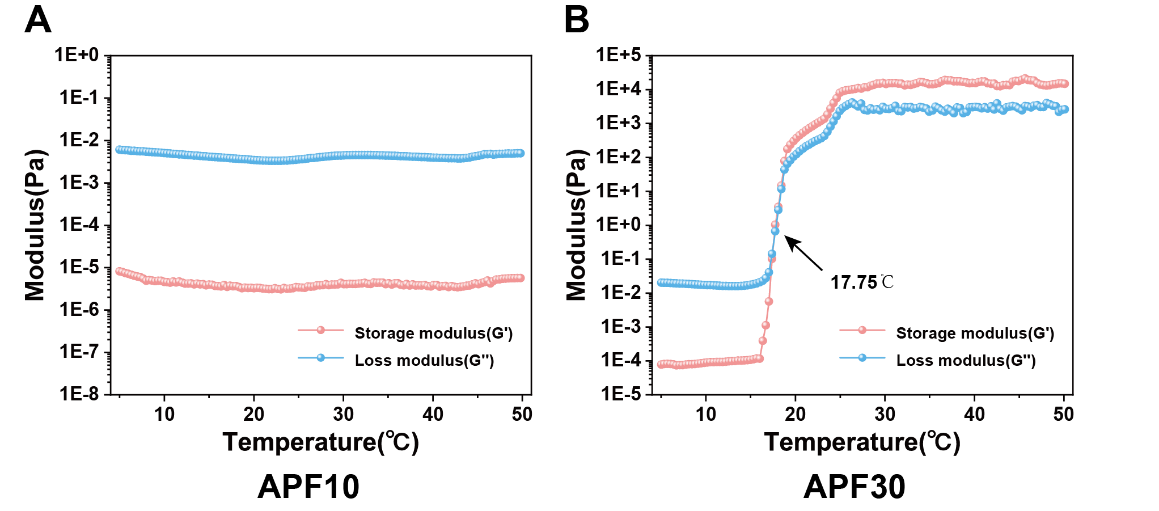


**Figure S4.** Temperature-dependent rheology of APF10 A) and APF30 B) hydrogel from 5 to 50℃.


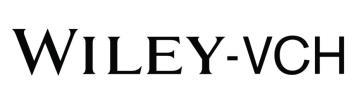


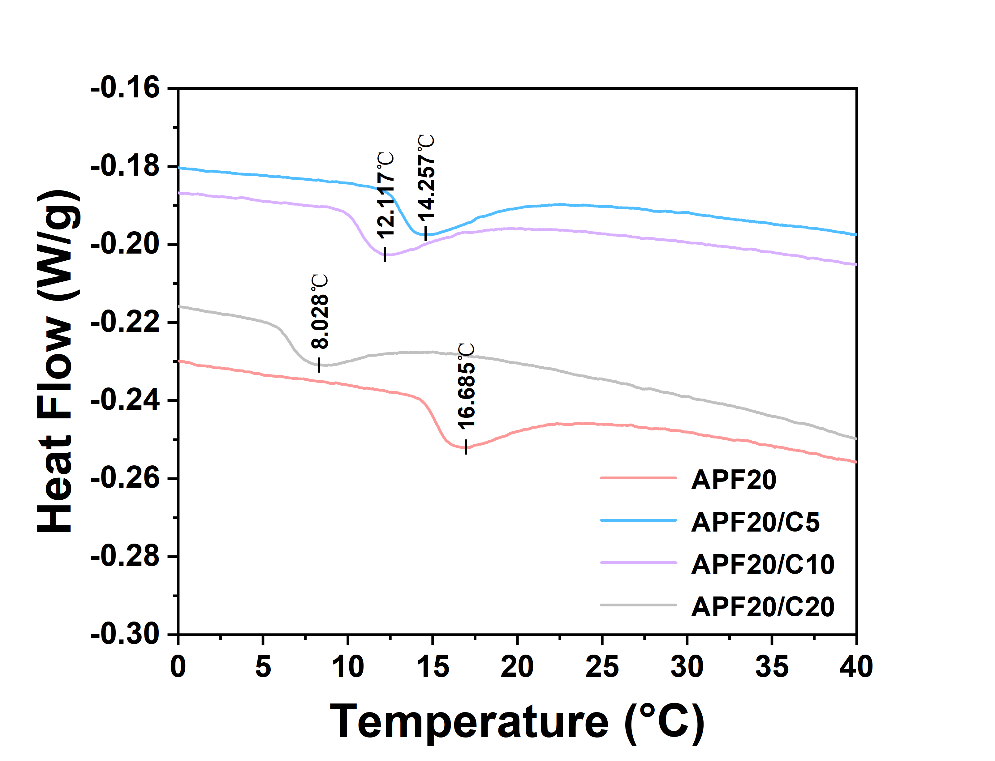


**Figure S5.** DSC thermograms of APF20, APF20/C5, APF20/C10 and APF20/C20 hydrogel.


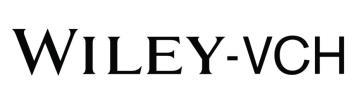


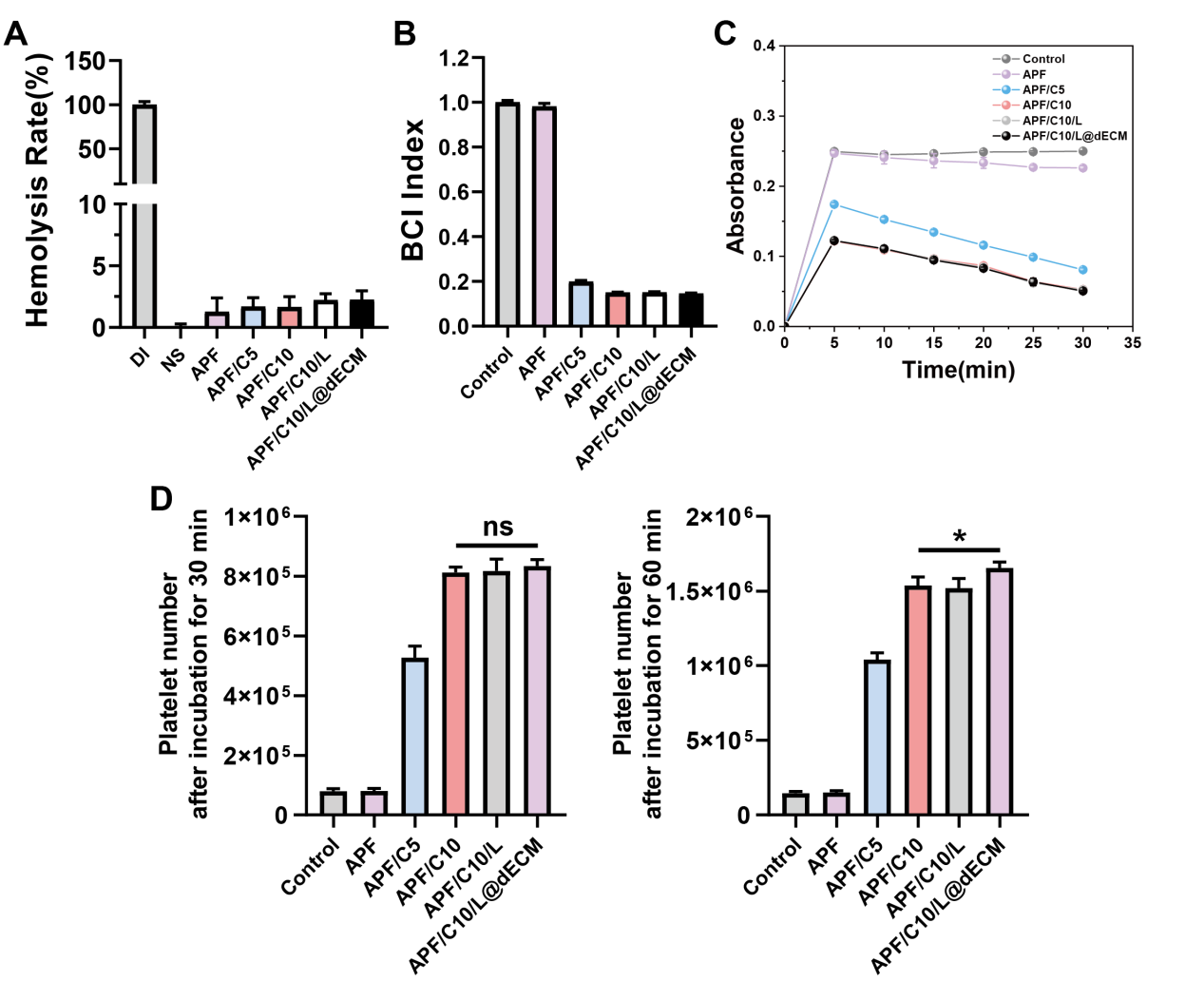


**Figure S6.** Evaluation of hydrogels biocompatibility and its procoagulant properties in vitro. A) Hemolysis test results. B) BCI comparison of different hydrogels. C) Dynamic coagulation curves of the different hydrogels. D) Platelet number on different hydrogels after incubation for 30 and 60 minutes. Data are expressed as the mean ± standard deviation (SD) (n=3). ns, not significant (P > 0.05), *P < 0.05.


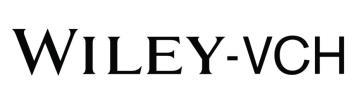


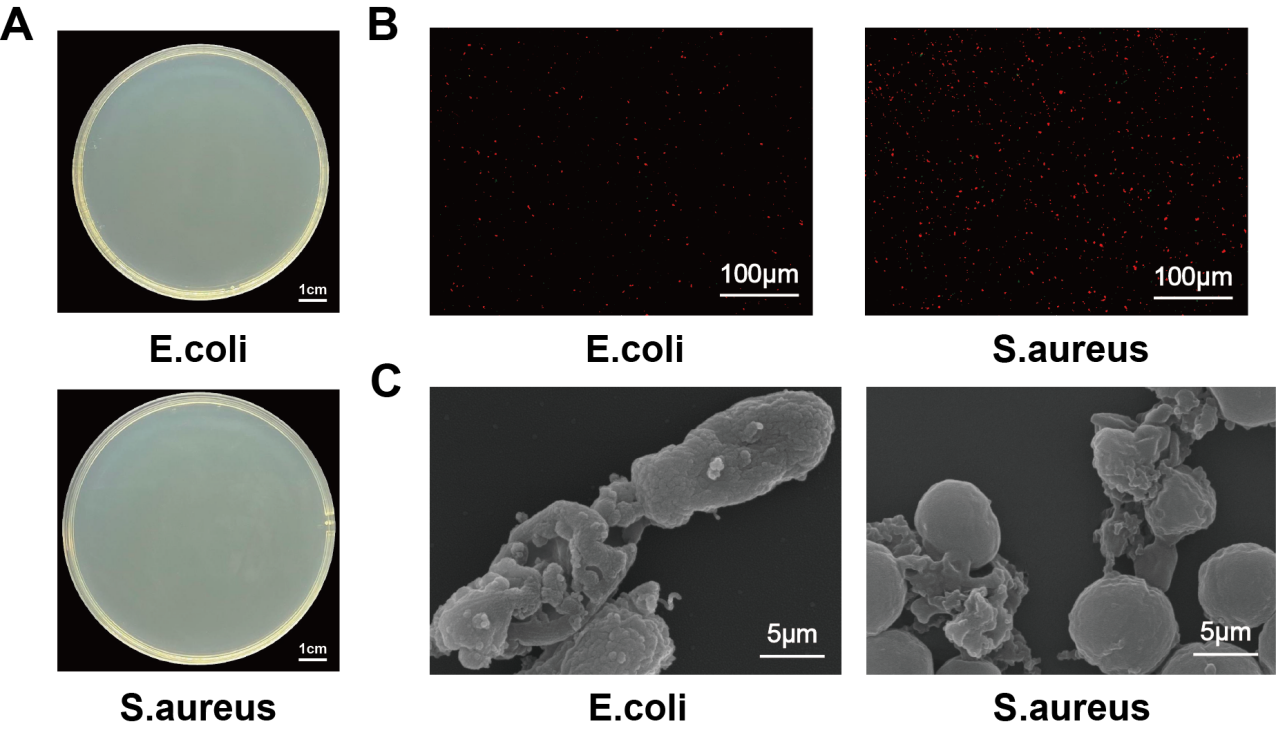


**Figure S7.** Evaluation of APF/C/L@dECM hydrogel antimicrobial properties in vitro. A) Colonization. B) Bacterial live-dead staining. The scale bar in images is 100 µm. C) SEM images of S. aureus and E. coli after treatment with APF/C/L@dECM hydrogel. The scale bar in images is 5 µm.


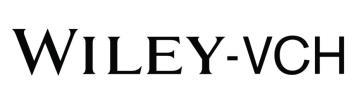


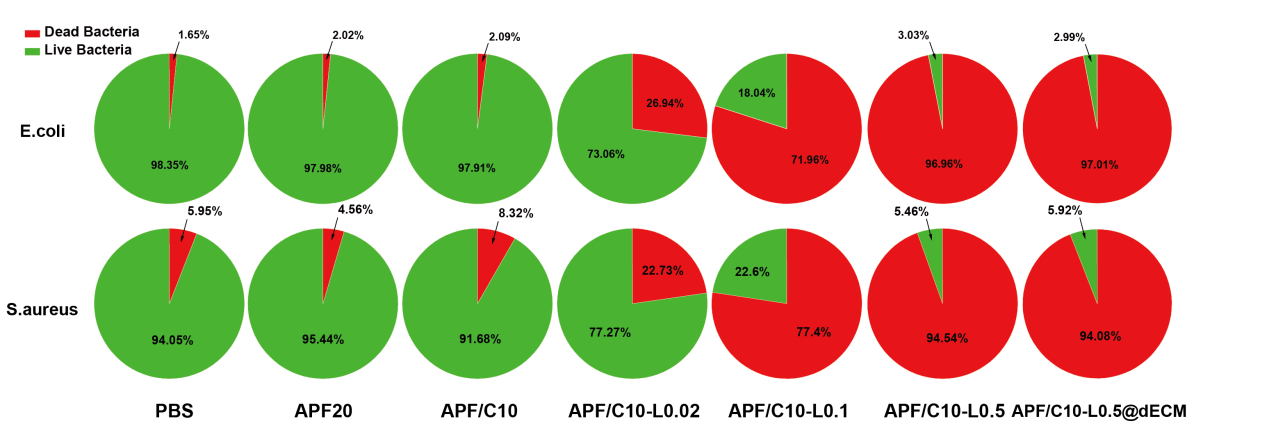


**Figure S8.** Semi-quantitative data of bacterial live-dead staining showing the different proportion live (green) and dead (red) bacteria after different treatments.


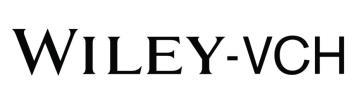


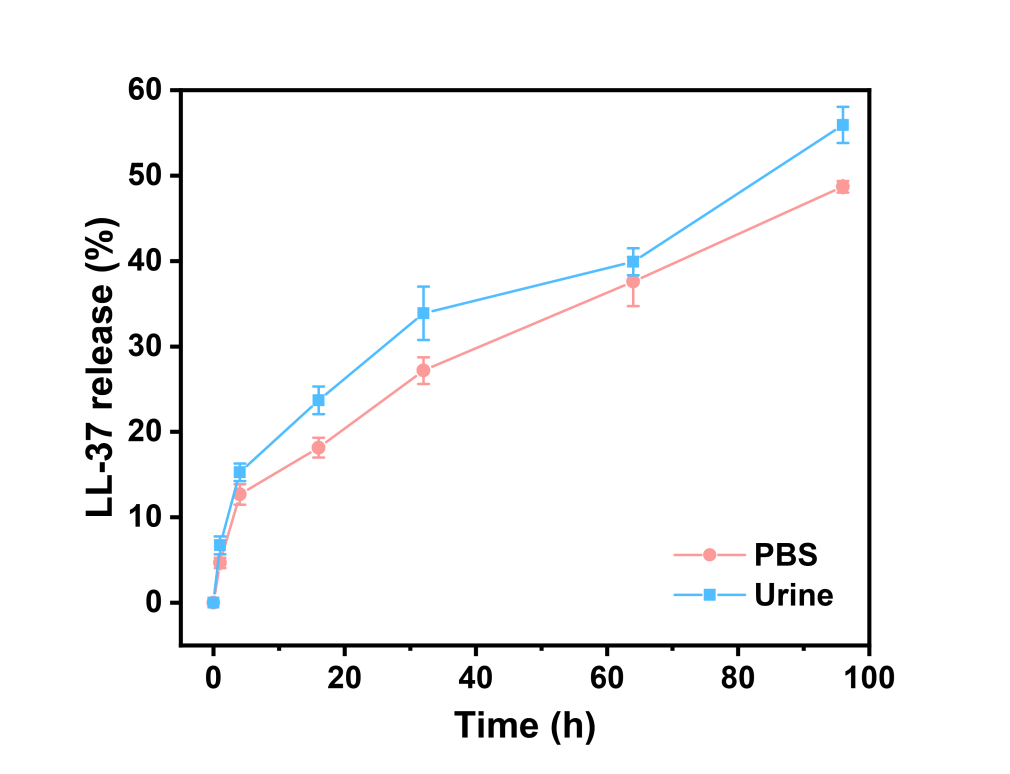


**Figure S9.** LL-37 antimicrobial peptide release rates of APF/C10/L-0.5 hydrogel in PBS and urine at 35℃ as a function of time. Data are expressed as the mean ± standard deviation (SD) (n=3).


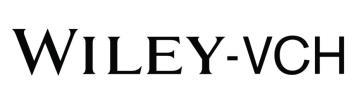


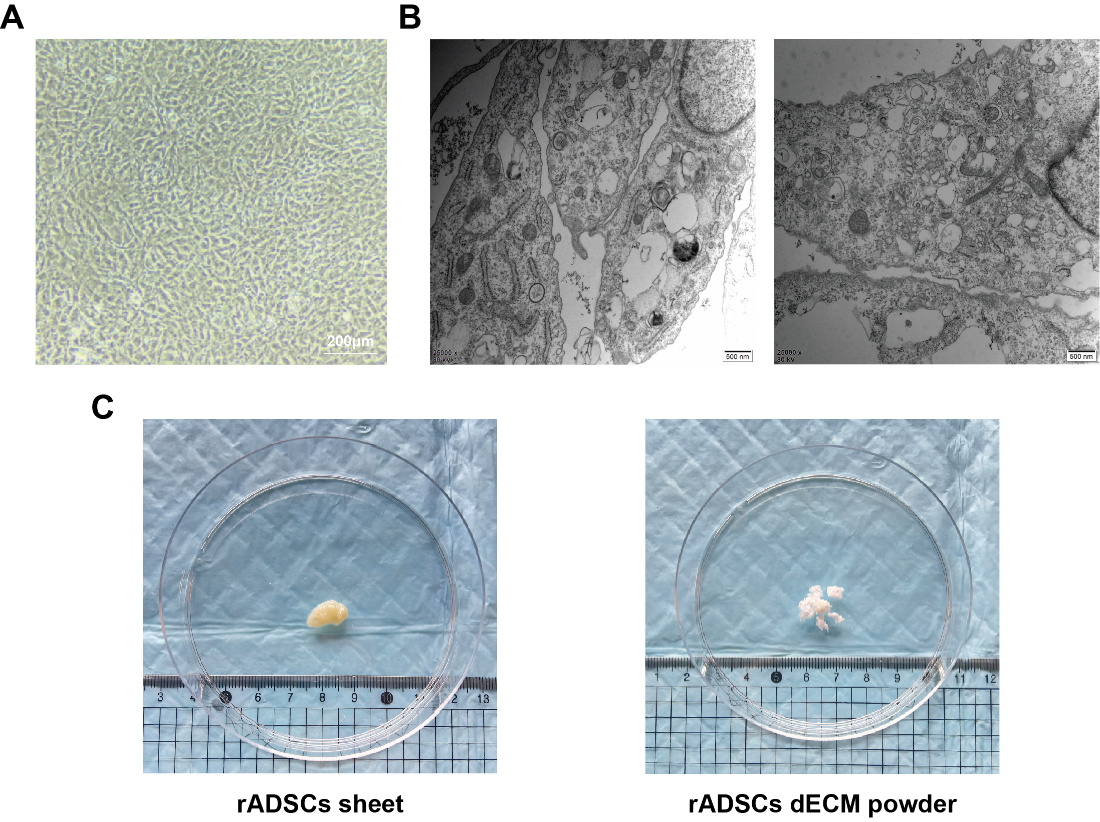


**Figure S10.** A) Bright-field images of ADSCs sheet. B) TEM images showing the ultrastructure of the ADSCs sheet, including a large number of tight junctions and gap links. C) Photograph of rADSCs sheet and rADSCs dECM powder.


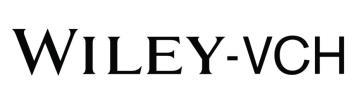


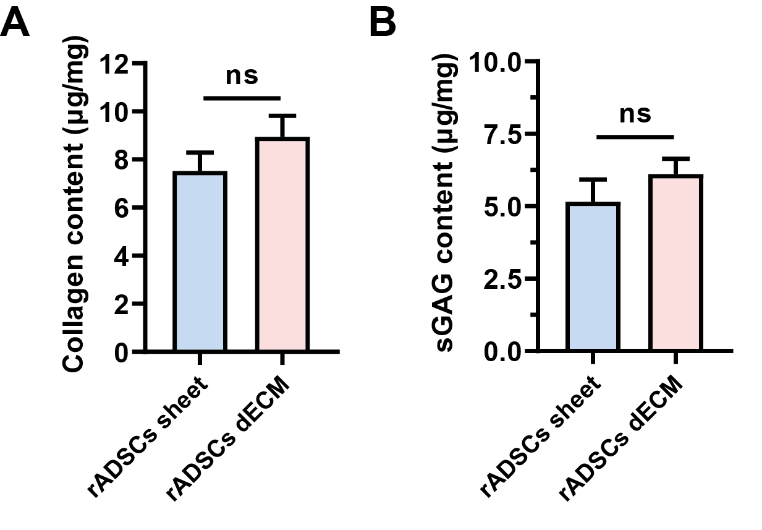


**Figure S11.** Comparison of collagen content A) and sGAG content B) in the rADSCs sheets and rADSCs dECM. Data are expressed as the mean ± standard deviation (SD) (n = 3).“ns” indicates P > 0.05, no statistically significant.


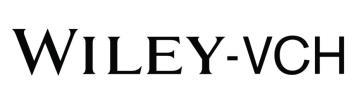


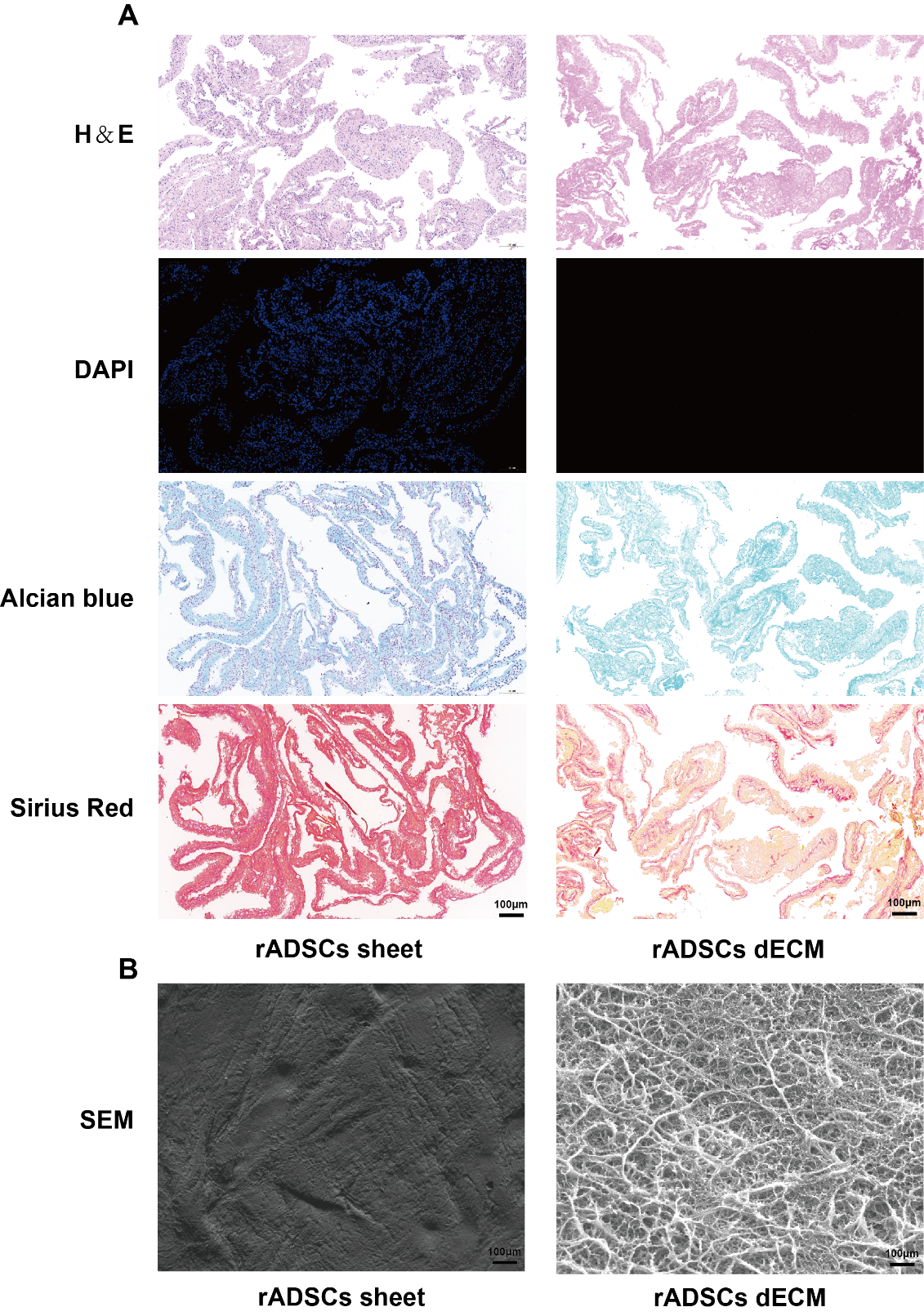


**Figure S12.** A) HE、DAPI、Alcian blue and Sirius Red staining of rADSCs sheets before and after decellularization. The absence of cell nuclei and remaining of collagen and glycosaminoglycan was observed in the decellularized sample. B) SEM of rADSCs sheets before and after decellularization.


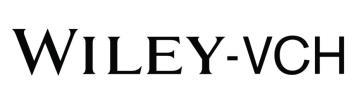


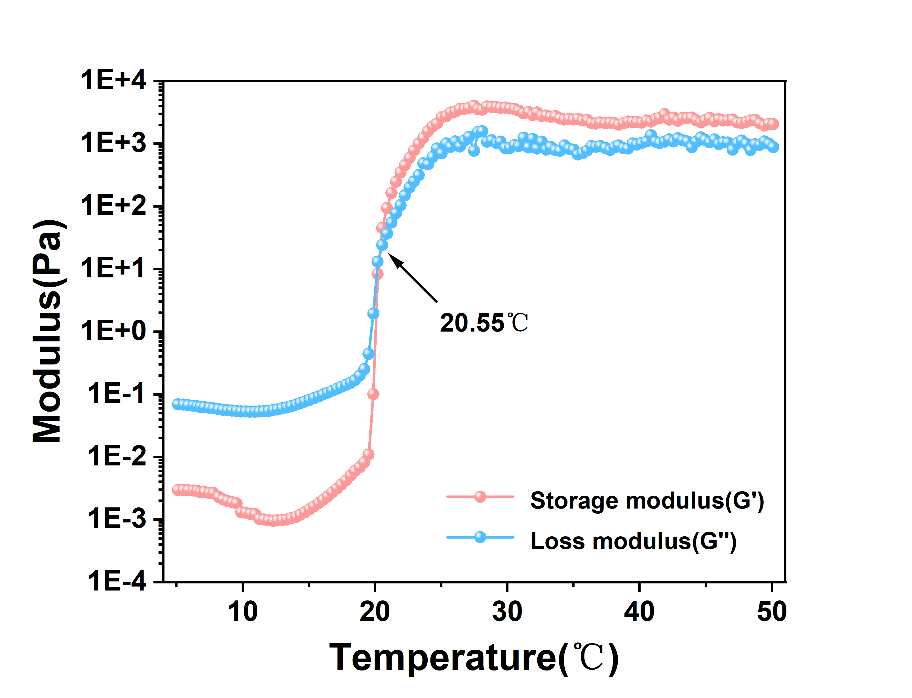


**Figure S13.** Temperature-dependent rheology of APF20/C10-L-0.5@dECM80 hydrogel from 5 to 50℃.


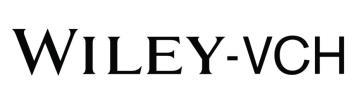


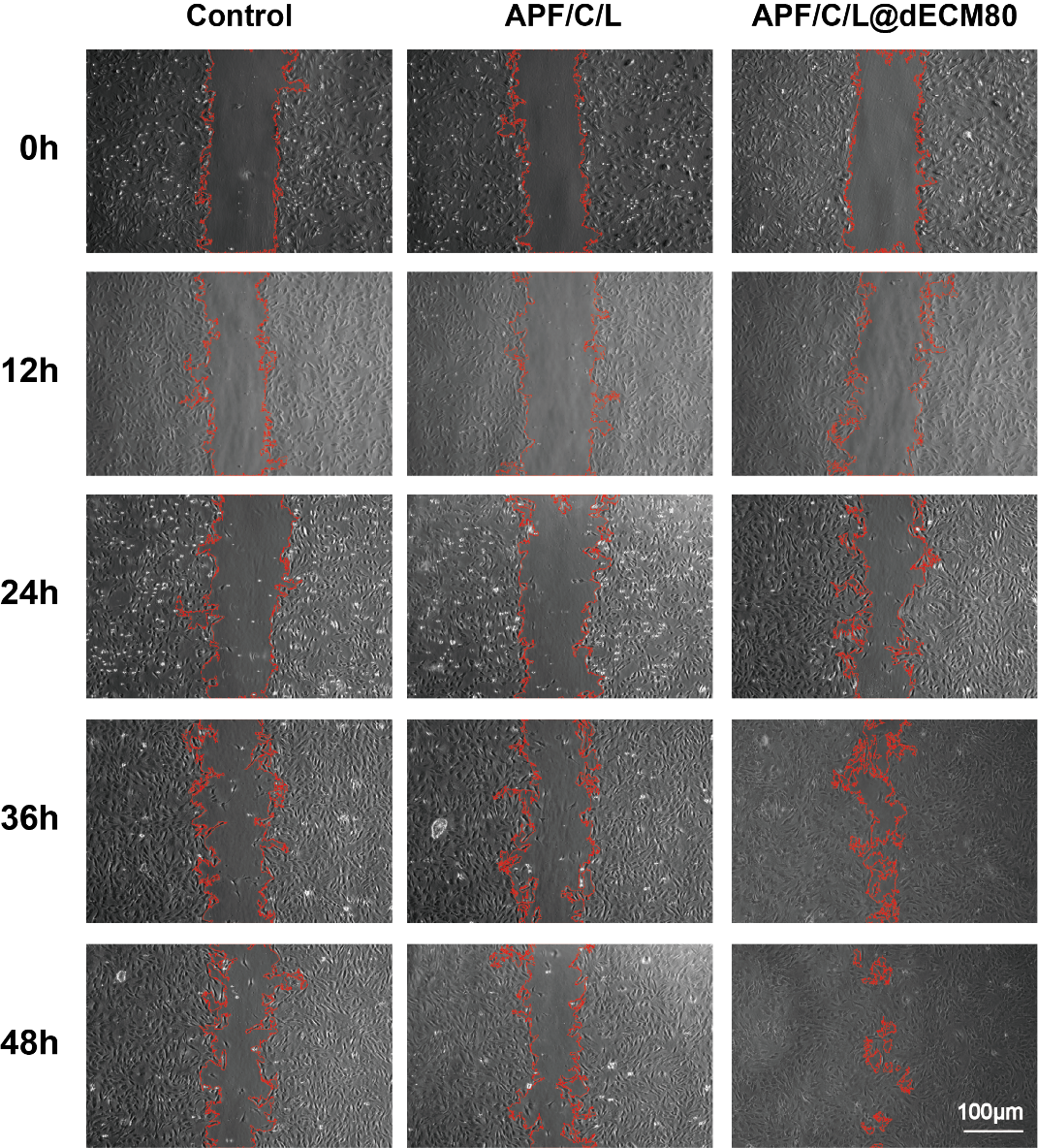


**Figure S14.** Bright-field image of ADSCs cultured under different conditions (PBS, APF20/C10-L-0.5 (APF/C/L) and APF20/C10-L-0.5@dECM 80 (APF/C/L@dECM80)) for 12h, 24h, 36h and 48h in wound healing experiments.


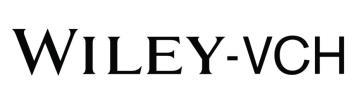


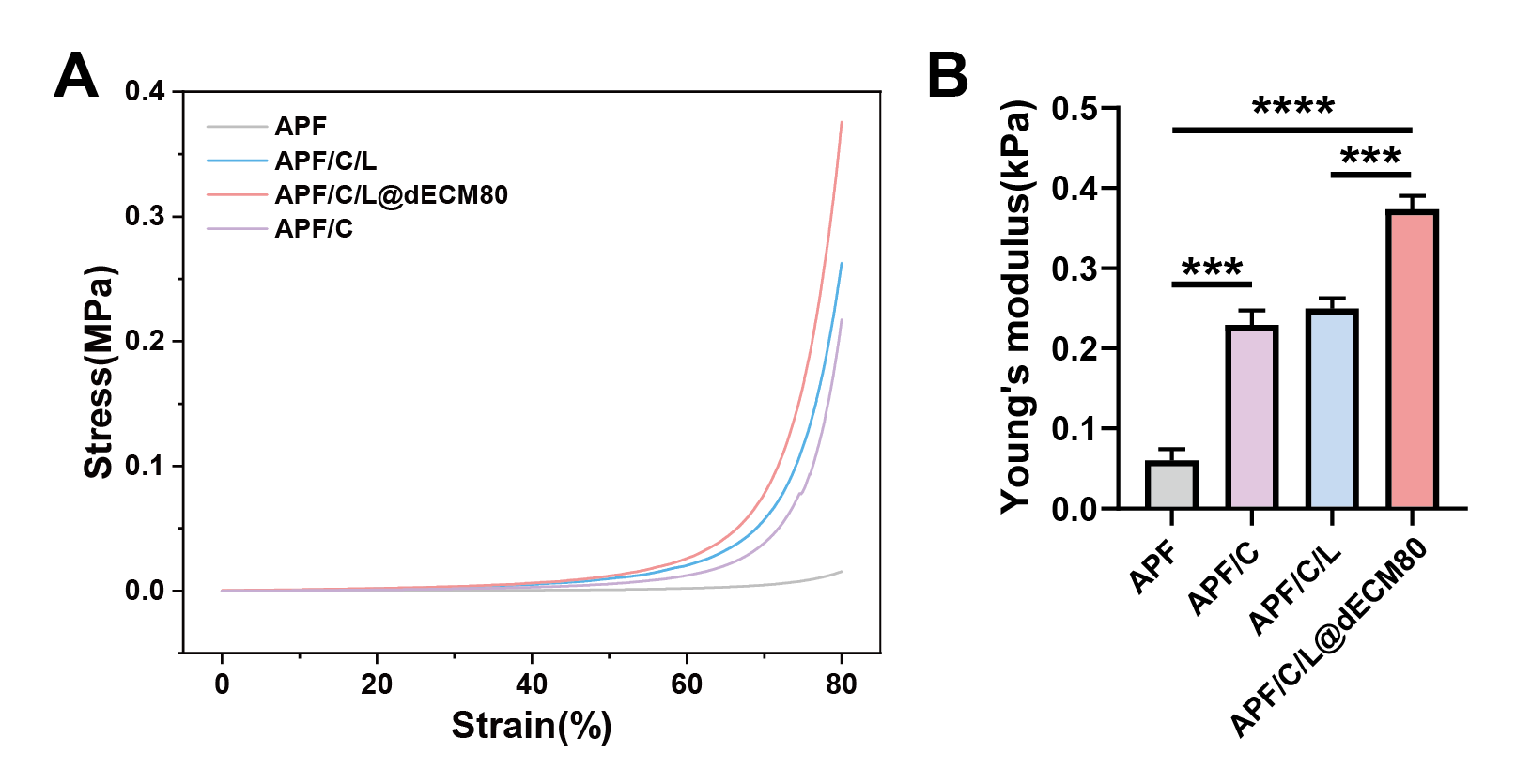


**Figure S15.** Compressive stress-strain curves A) and Young's modulus B) of hydrogels with different components. Data are expressed as the mean ± standard deviation (SD) (n = 3). ***P < 0.001, ****P < 0.0001.


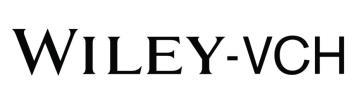


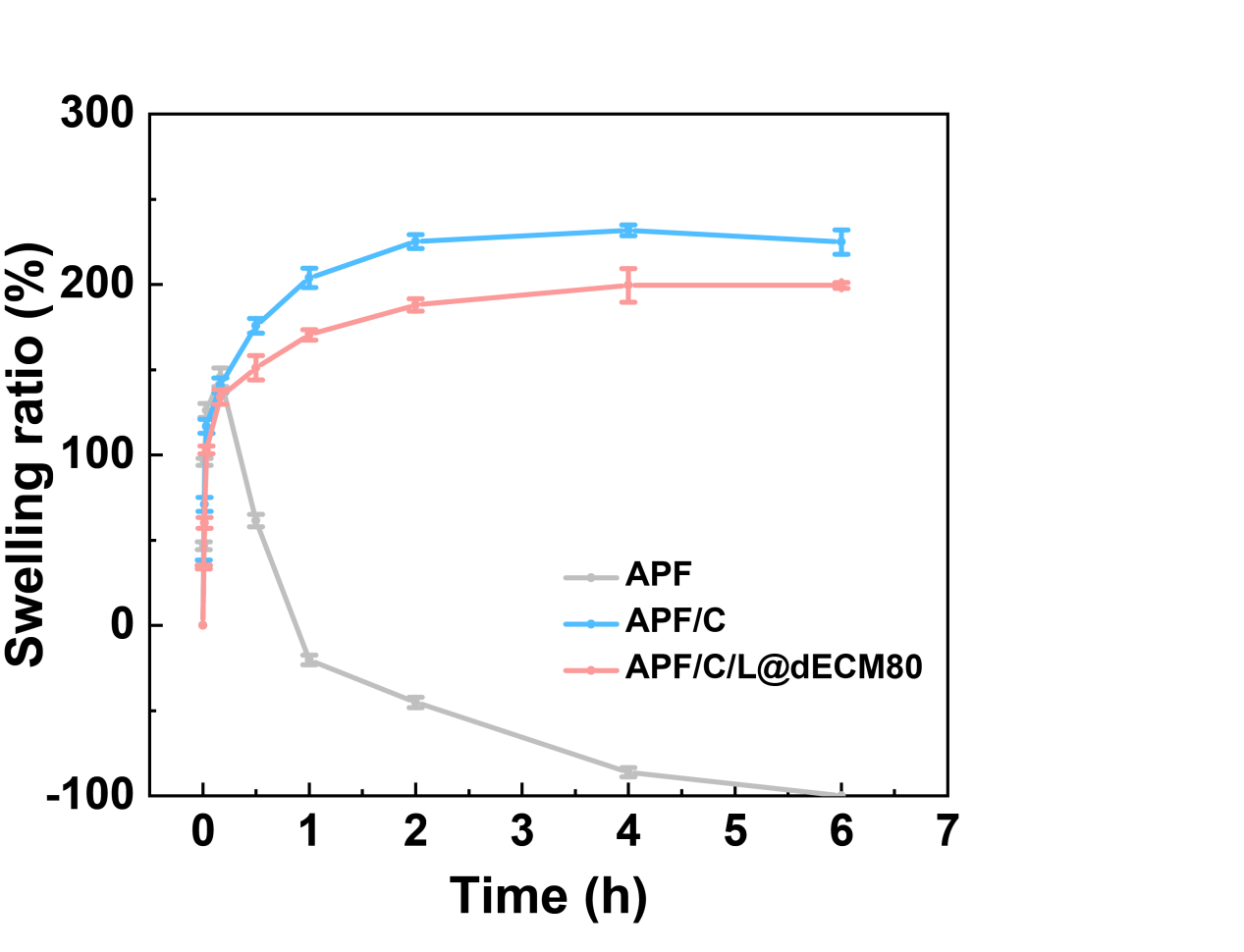


**Figure S16.** Swelling ratio of above three hydrogels in PBS at 35℃ as a function of time. Data are expressed as mean ± standard deviation (SD) (n =3).


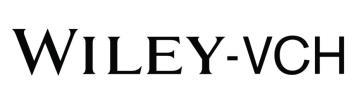


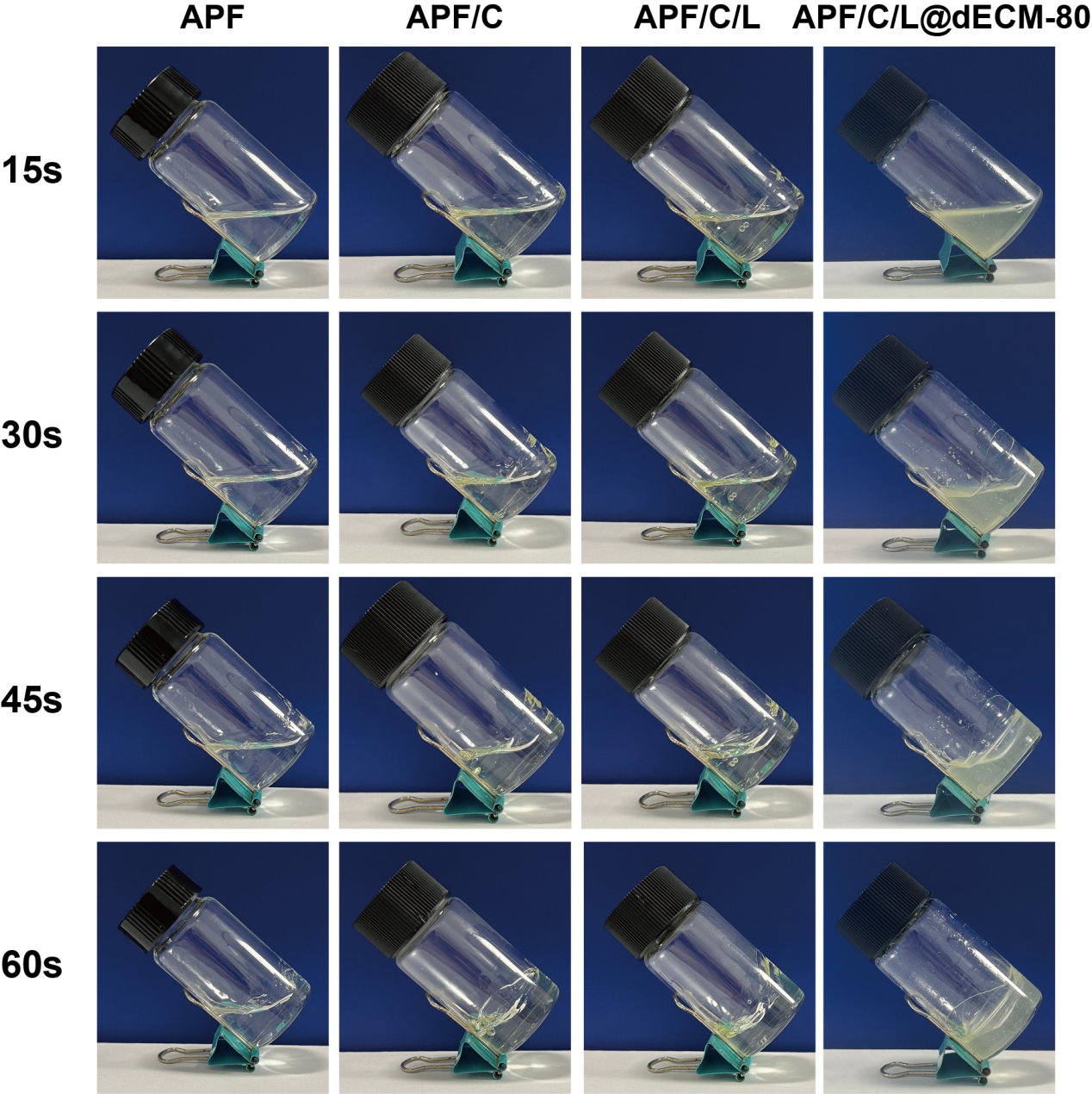


**Figure S17.** In vitro solution-gel transformation time of different groups of hydrogels at 35°C assessed by the tilted tube method.


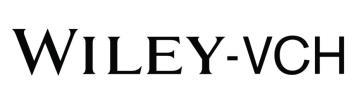


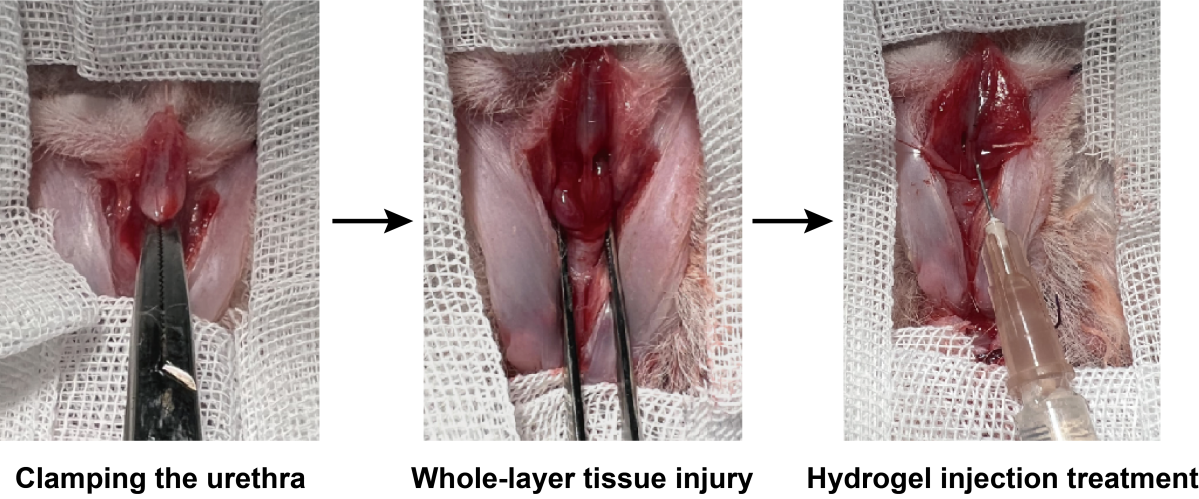


**Figure S18.** Diagram of whole-layer tissue injury of the urethra and the procedure of hydrogel injection treatment.


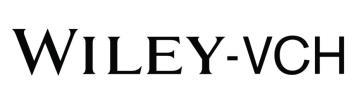


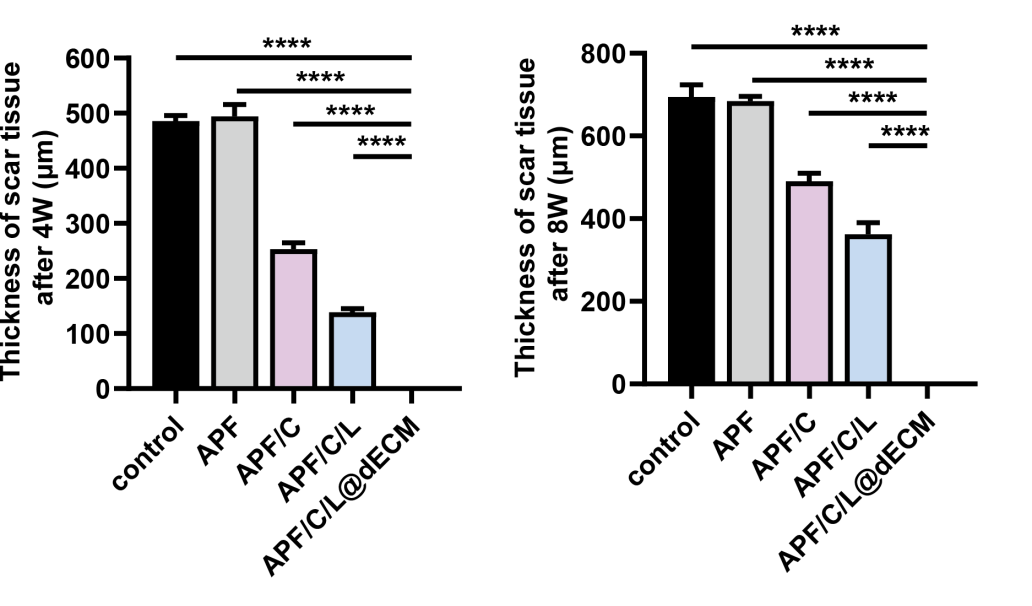


**Figure S19.** Comparison of scar tissue thickness formed in different groups at 4- A) and 8-weeks B) post-injury. Data are expressed as mean ± standard deviation (SD) (n =3). ****P < 0.0001.


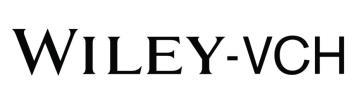


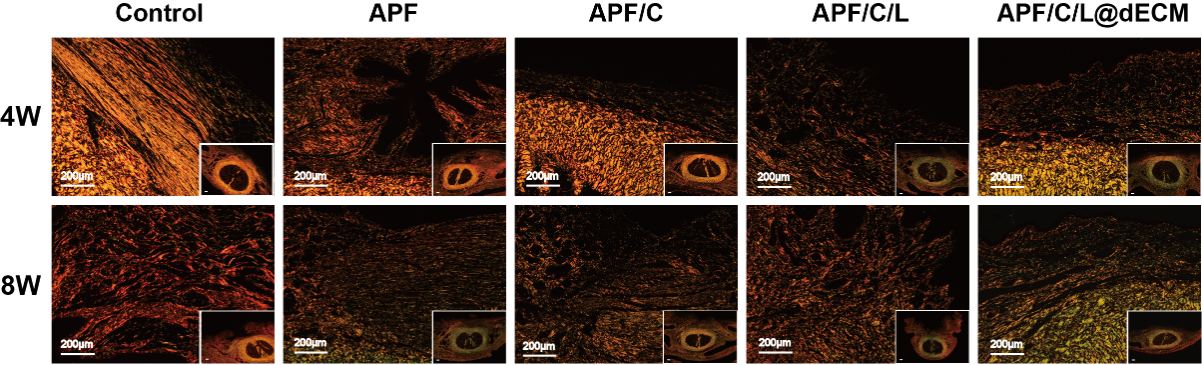


**Figure S20.** Sirius red staining of the urethra wounds after treatment at 4W and 8W under polarized light microscopy. The inset is the same area imaged at low magnification. The scale bar in both the high and low magnification images is 200 µm.


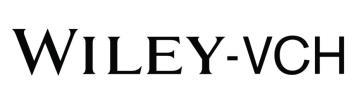


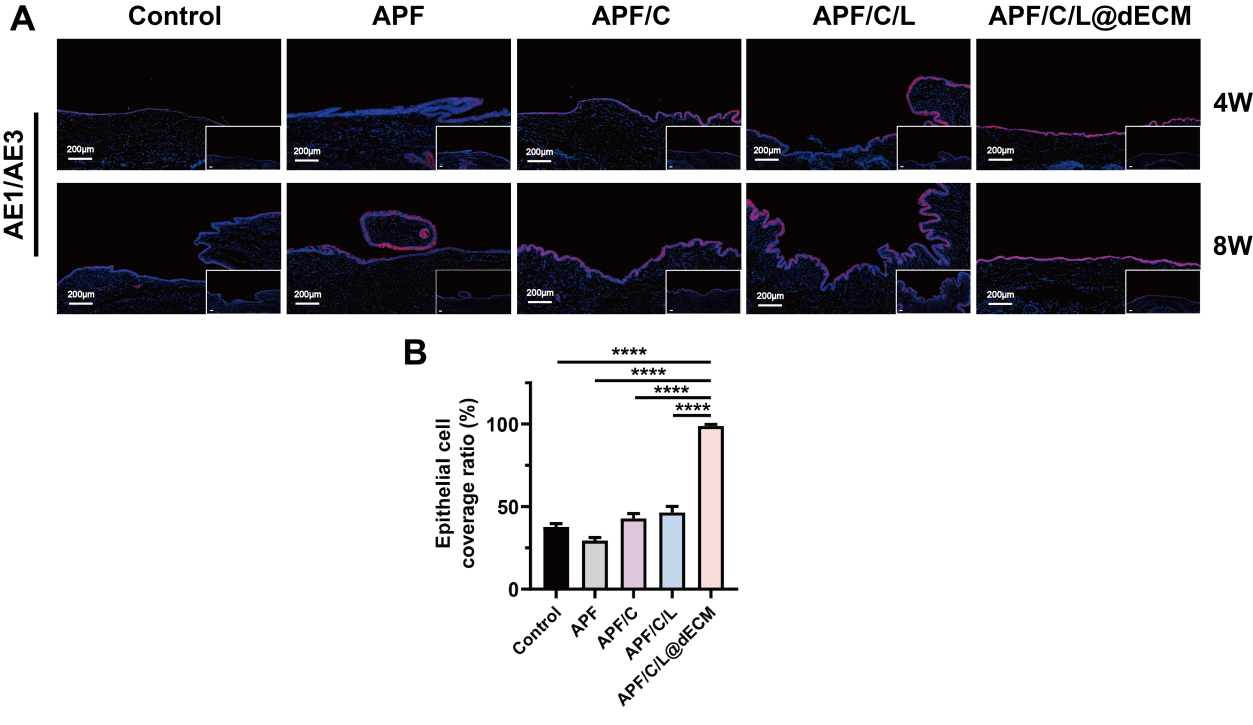


**Figure S21.** A) Immunofluorescence images of epithelial tissue (AE1/AE3); blue represents the nucleus, red represent AE1/AE3 markers. The inset is the same area imaged at low magnification. The scale bar in both the high and low magnification images is 200 µm. B) Analysis of the epithelial cell coverage ratio in the urethral wounds was used to assess the regeneration efficiency of epithelial tissue after 4 weeks of treatment with (i.e., control, APF, APF/C, APF/C/L, and APF/C/L@dECM). Data are expressed as the mean ± standard deviation (SD) (n = 3). ****P < 0.0001.


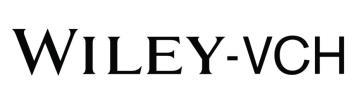


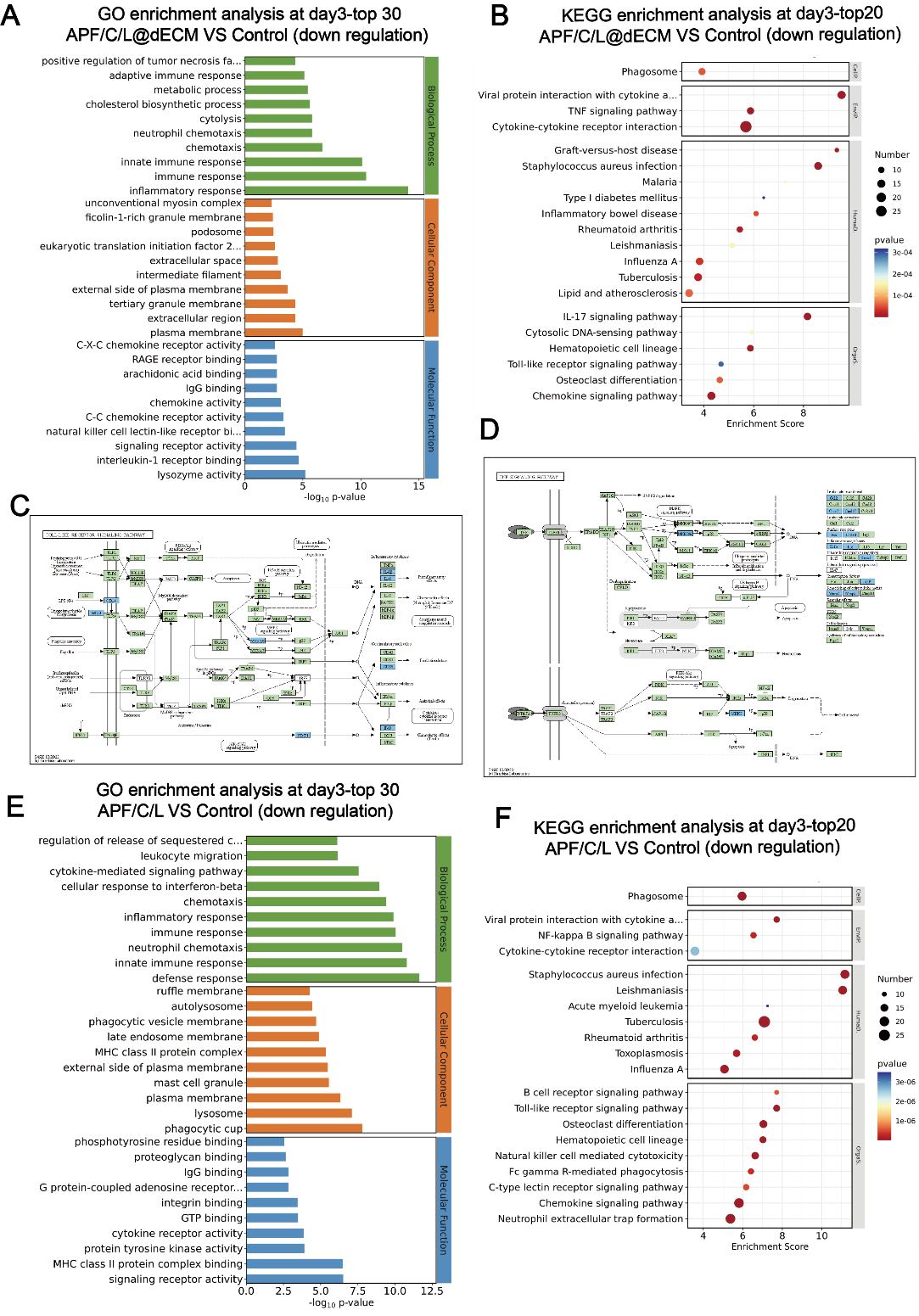


**Figure S22.** A) GO enrichment analysis of the downregulated genes in the top 30 at day 3 between Control and APF/C/L@dECM group. B) KEGG enrichment analysis of the downregulated genes in the top 20 at day 3 between Control and APF/C/L@dECM group. Toll-like receptor signaling pathway C) and TNF signaling pathway D) maps between Control and APF/C/L@dECM group. E) GO enrichment analysis of the downregulated genes in the top 30 at day 3 between Control and APF/C/L group. F)KEGG enrichment analysis of the downregulated genes in the top 20 at day 3 between Control and APF/C/L group.


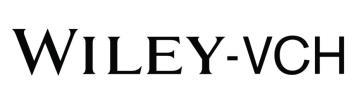


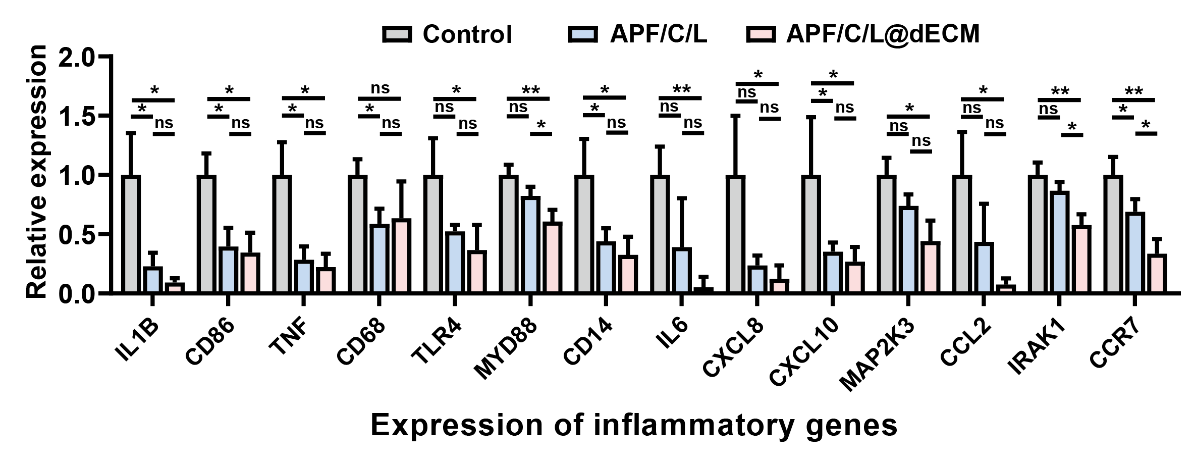


**Figure S23.** The expression of key inflammatory genes in different groups.


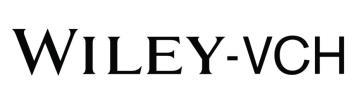


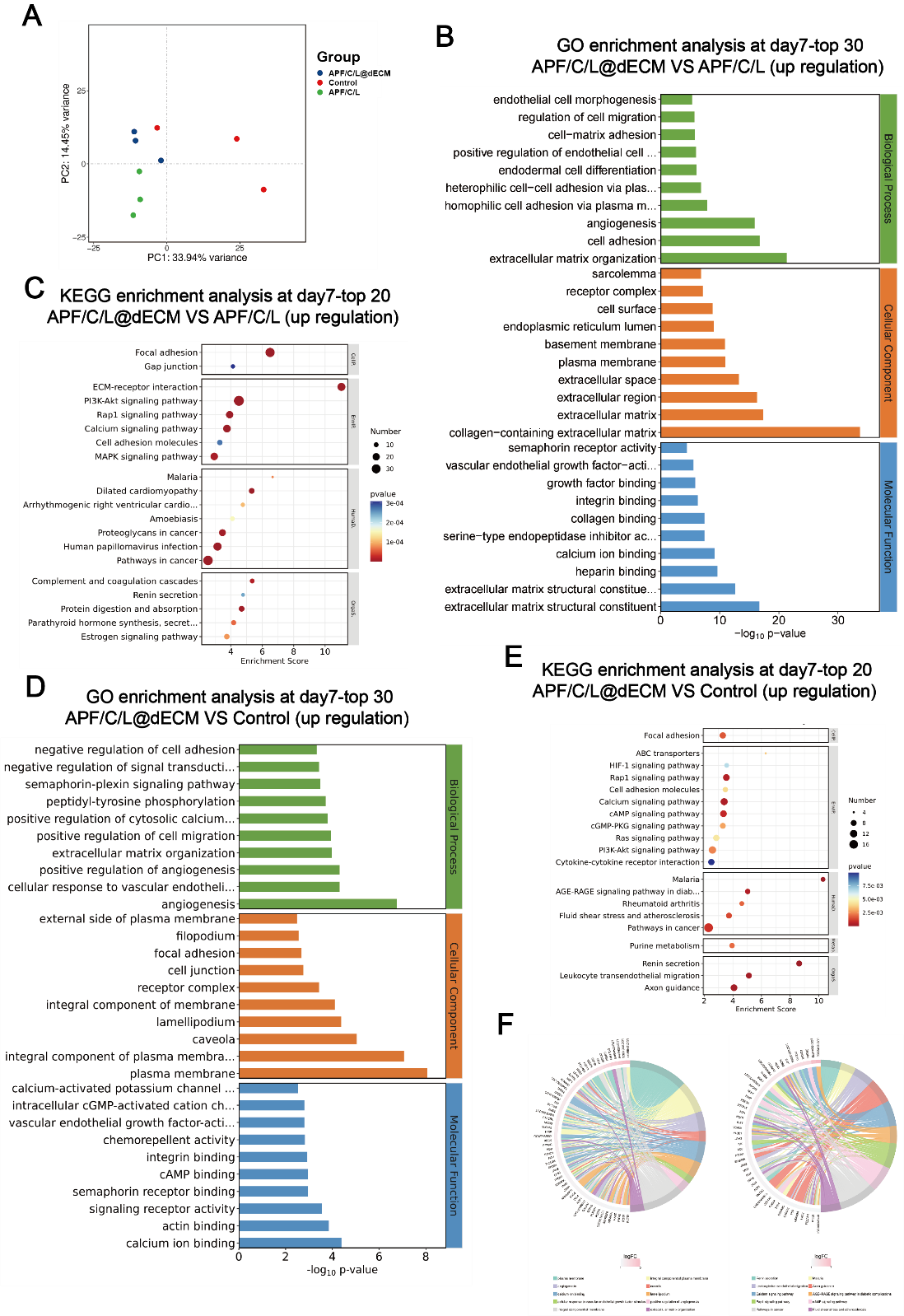


**Figure S24.** A) Principal components analysis (PCA) of each group of samples. B-C) GO and KEGG enrichment analysis of the upregulated genes at day 7 between APF/C/L and APF/C/L@dECM group. D-F) GO and KEGG enrichment analysis of the upregulated genes at day 7 between control and APF/C/L@dECM group.


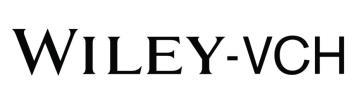


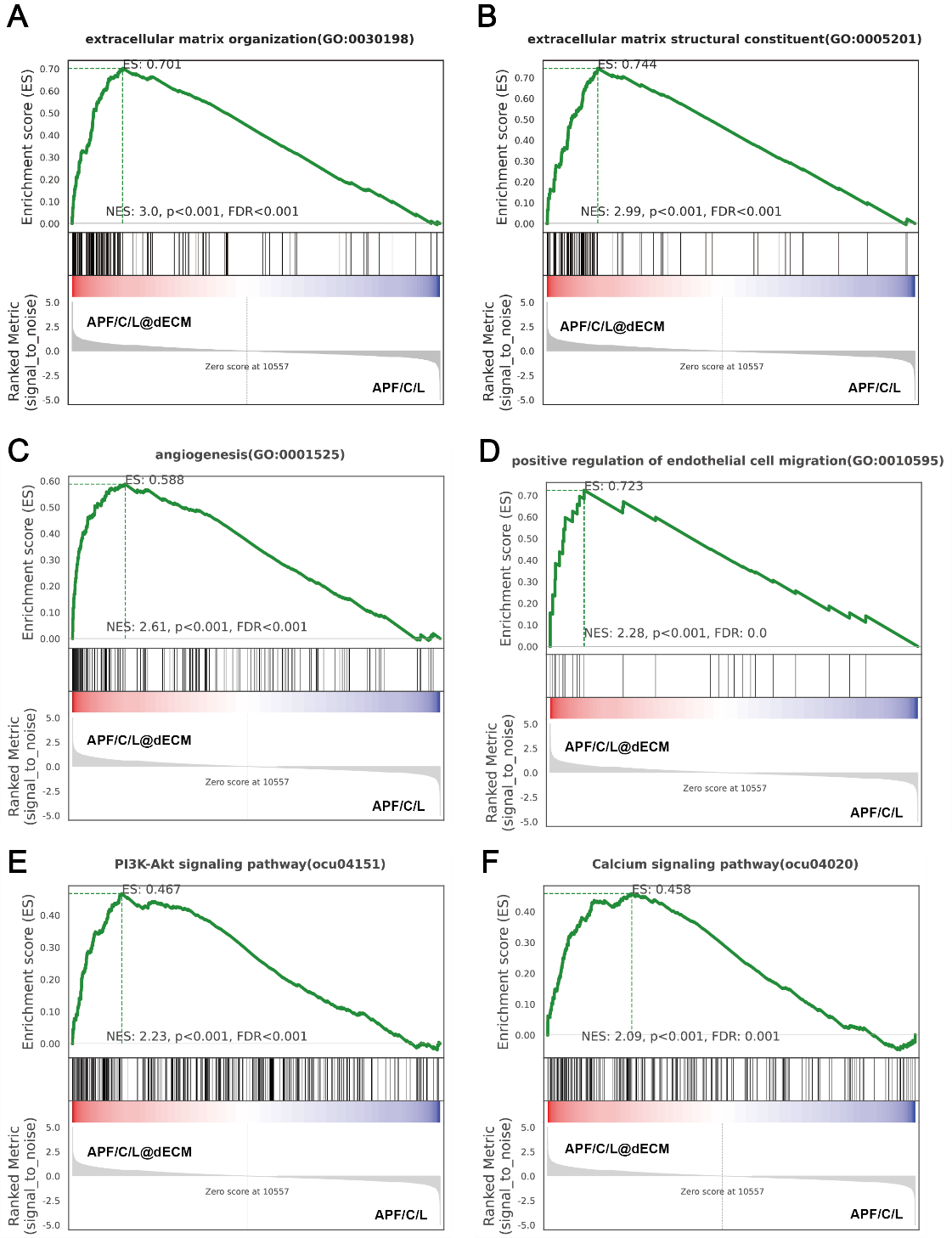


**Figure S25.** Gene Set Enrichment Analysis (GSEA) confirmed that the expression of pathways related to angiogenesis and tissue regeneration was increased in the APF/C/L@dECM group. A) Extracellular matrix organization. B) Extracellular matrix structural constituent. C) Angiogenesis. D) Positive regulation of endothelial cell migration. E) PI3k-Akt signaling pathway. F) Calcium signaling pathway.


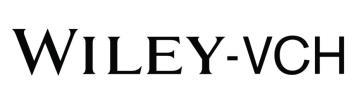


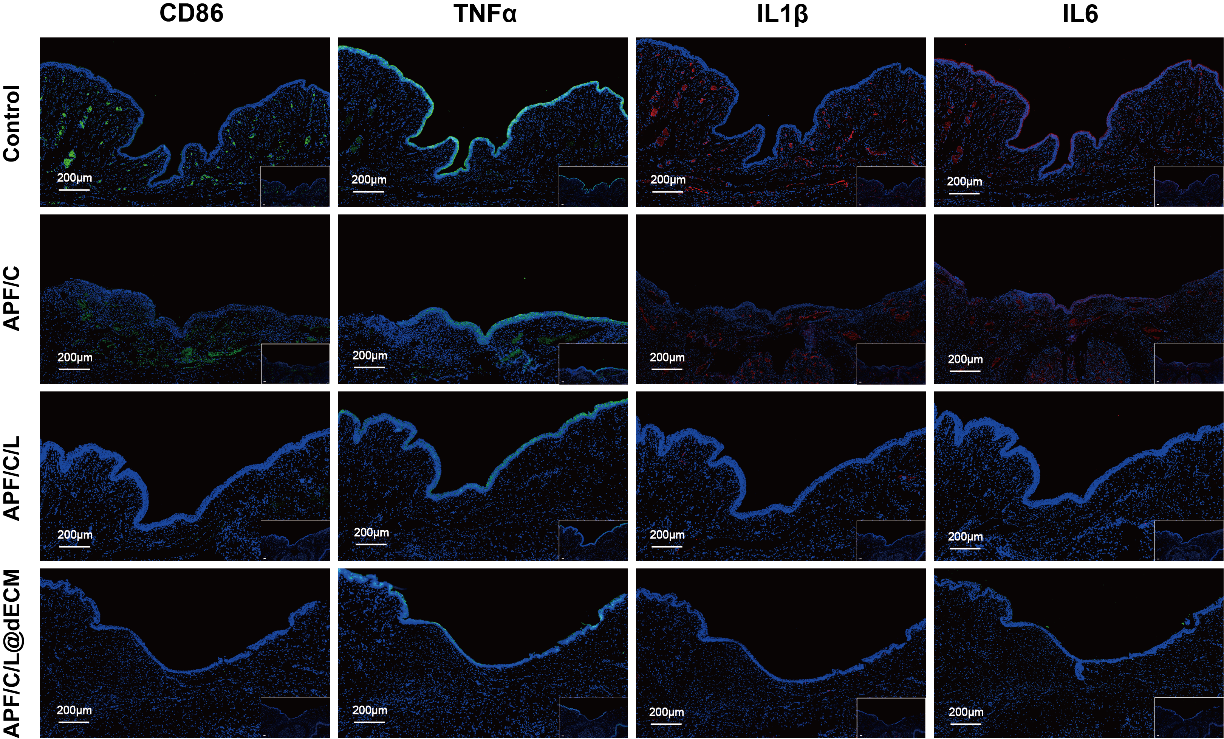


**Figure S26.** Immunofluorescence images of CD86、TNFA、IL1β and IL6 of urethral wounds after 3 days under different treatments. Blue represents the nucleus, red and green represent the corresponding markers, and the inset is a low magnification (scale bar in both magnification and inset: 200 µm).


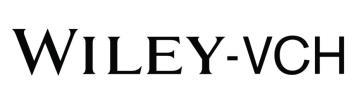


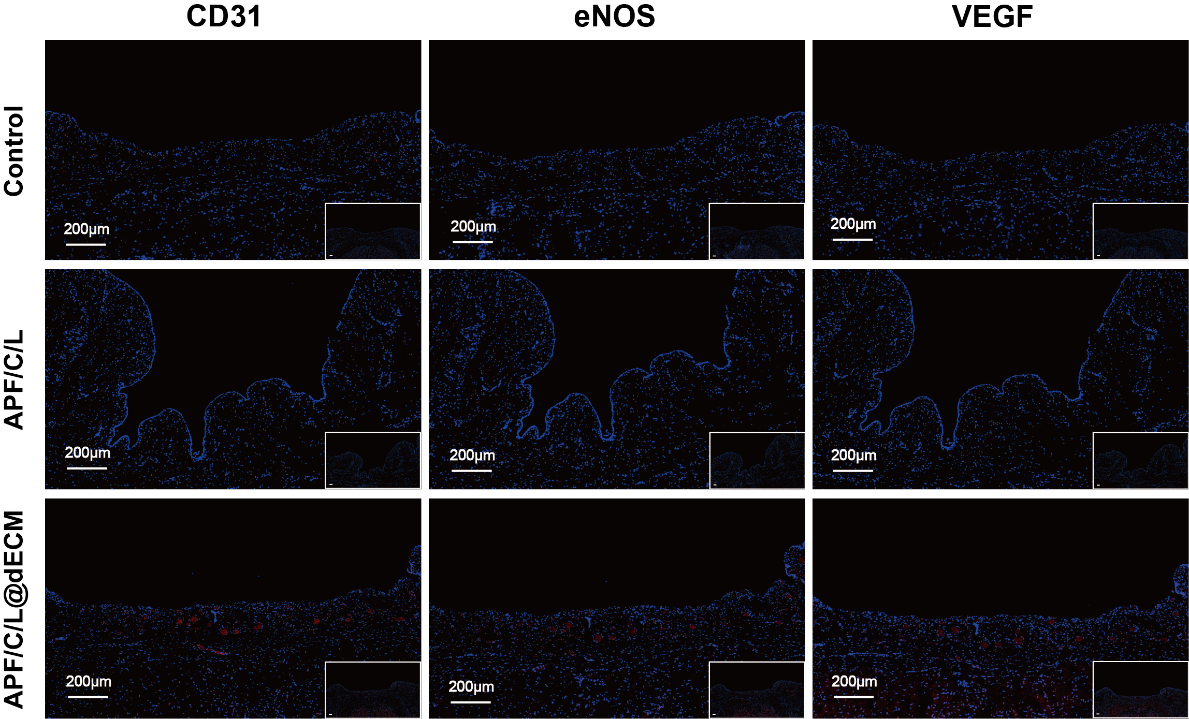


**Figure S27.** Immunofluorescence images of CD31、eNOS and VEGF of urethral wounds after 7 days under different treatments. Blue represents the nucleus, red represent the corresponding markers, and the inset is a low magnification (scale bar in both magnification and inset: 200 µm).


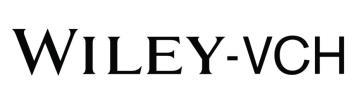


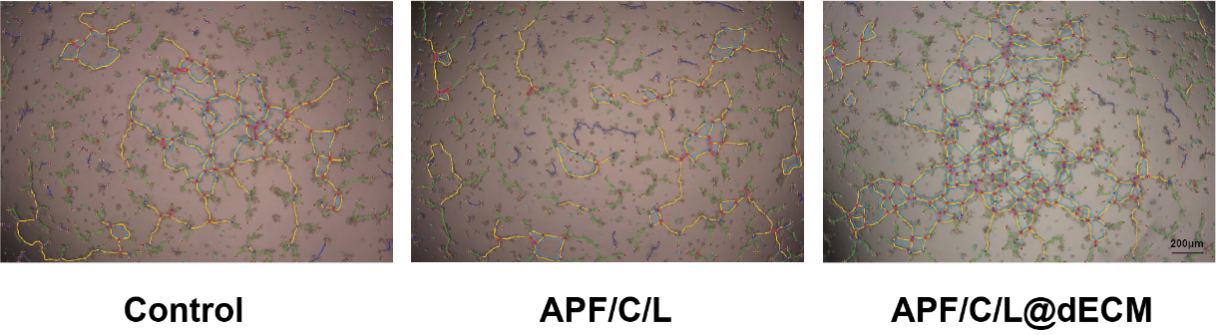


**Figure S28.** The tube formation ability of HUVECs with different treatments.


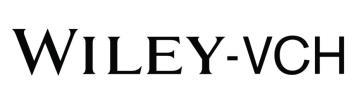


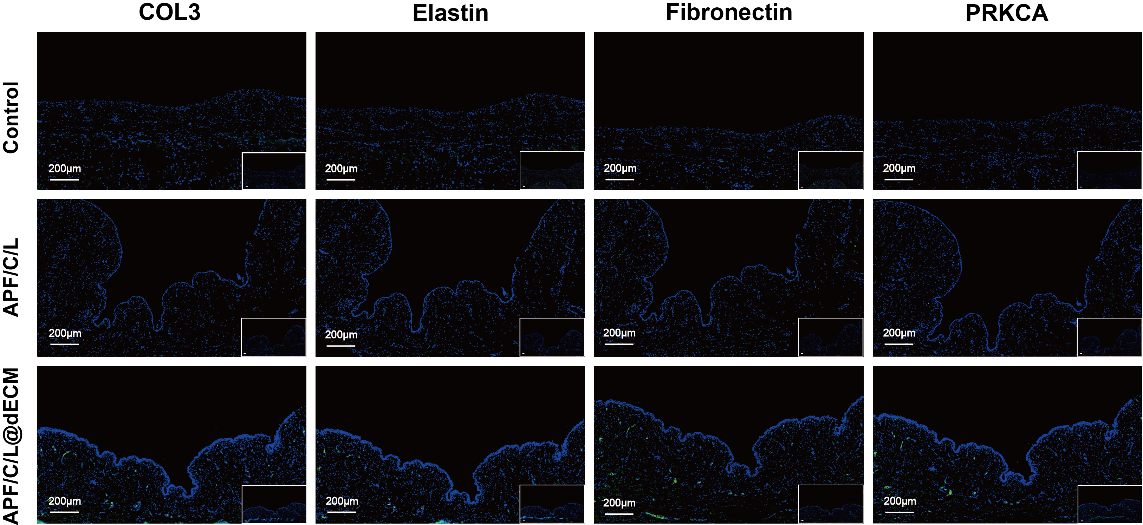


**Figure S29.** Immunofluorescence images of COL3、ELN、FN and PRKCA of urethral wounds after 7 days under different treatments. Blue represents the nucleus, green represent the corresponding markers, and the inset is a low magnification (scale bar in both magnification and inset: 200 µm).


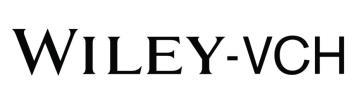


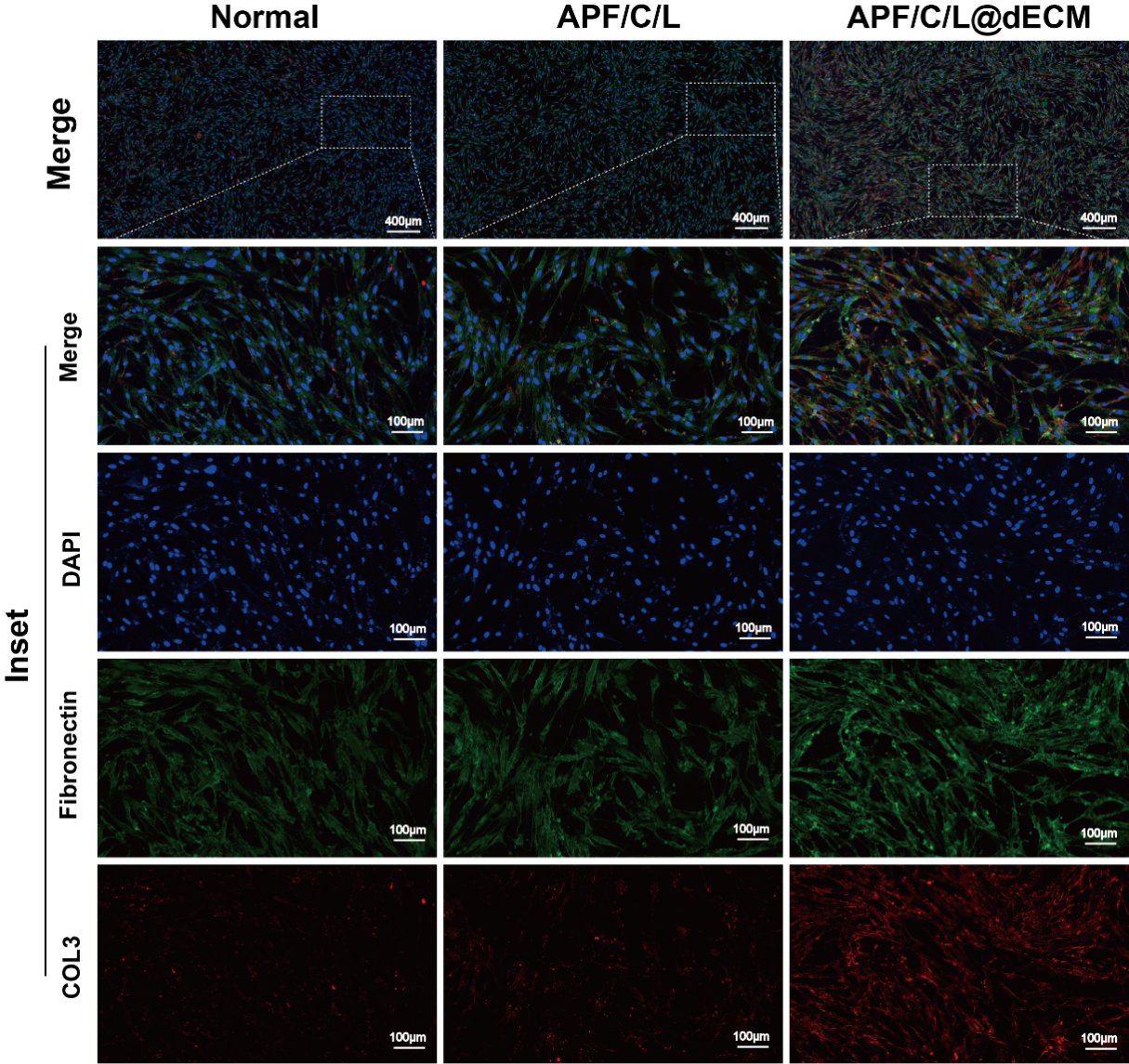


**Figure S30.** Immunofluorescence images of COL3 and FN of HFF after 7 days under different treatments. Blue represents the nucleus, green represent the corresponding marker. The scale bar in images is 100 or 400 µm.


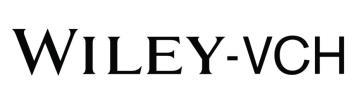


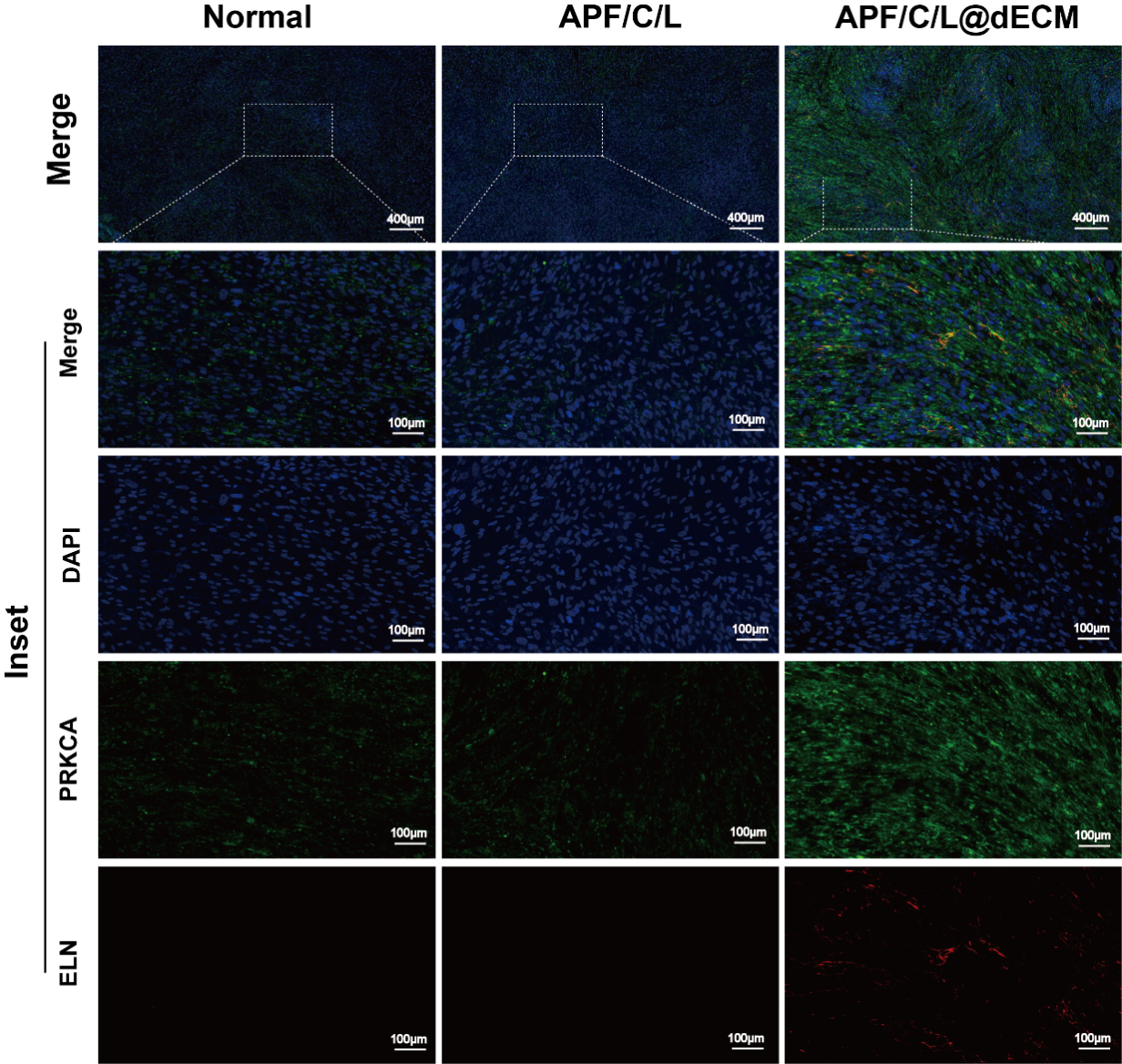


**Figure S31.** Immunofluorescence images of ELN and PRKCA of HFF after 7 days under different treatments. Blue represents the nucleus, green represent the corresponding marker. The scale bar in images is 100 or 400 µm.


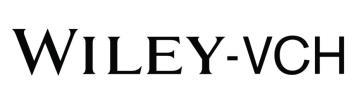


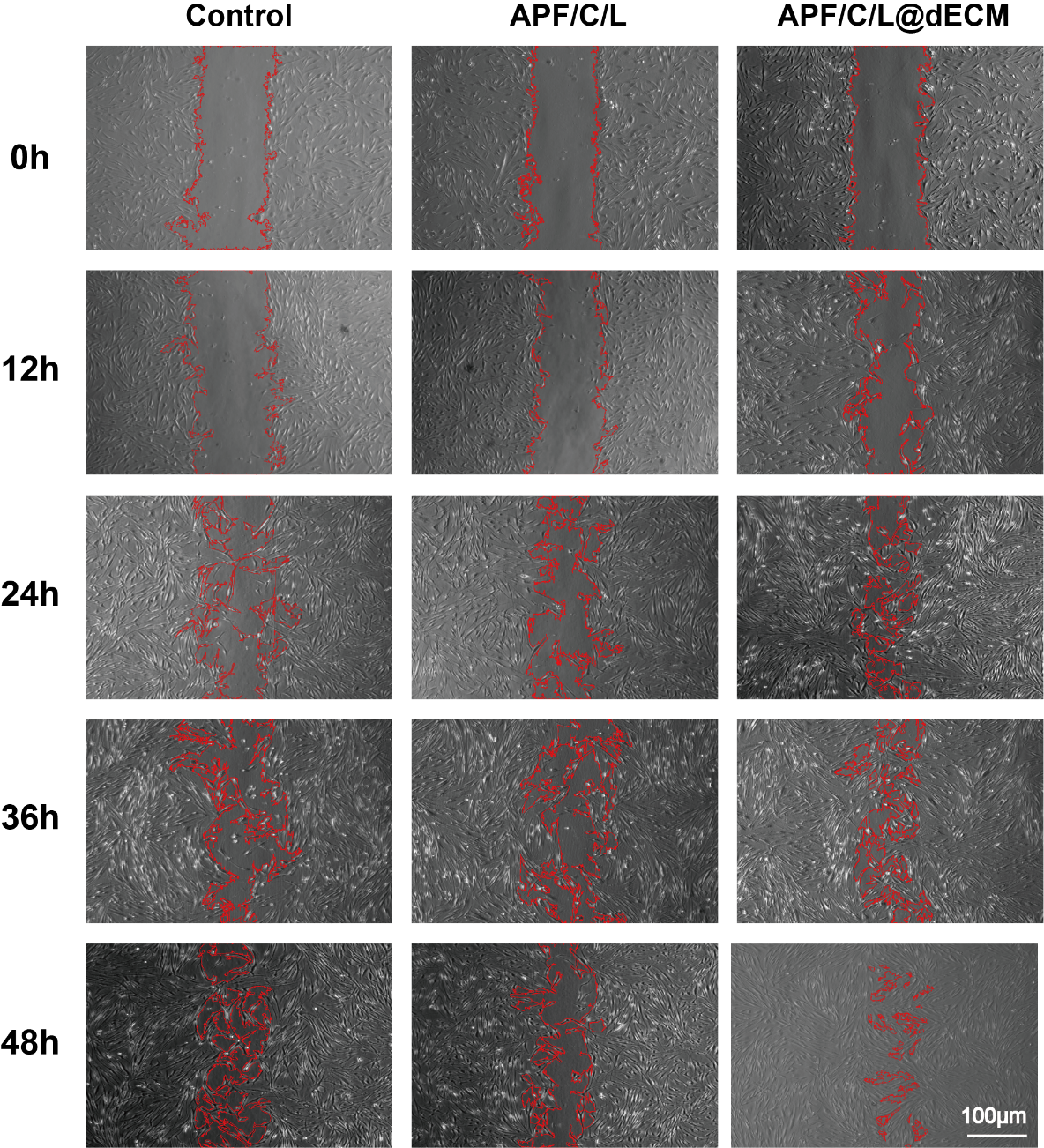


**Figure S32.** Bright-field image of HFF cultured under different conditions (PBS, APF/C/L and APF/C/L@dECM for 12h, 24h, 36h and 48h in wound healing experiments.


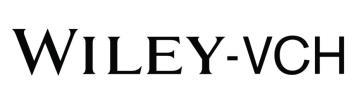


1. **Supplementary tables**

**Table S1.** Antibody provider and Catalog Number

| **Antibody** | **Provider** | **Catalog** | **Antibody** | **Provider** | **Catalog** |
| --- | --- | --- | --- | --- | --- |
| CD14 | Abcam | ab183322 | CD31 | Abcam | ab76533 |
| TLR4 | Abcam | ab13556 | PCNA | Abcam | ab29 |
| MYD88 | Abcam | ab133739 | α-SMA | Cell Signaling Technology | 48938 |
| IRAK1 | Abcam | ab302554 | COL1 | Abcam | ab138492 |
| MEK3 | Abcam | ab195037 | PRKCA | Abcam | ab32376 |
| CD68 | Abcam | ab283654 | ELN | ABclonal | A22733 |
| CD86 | Abcam | ab239075 | FN | Abcam | ab2413 |
| TNFα | Abcam | ab183218 | VEGFA | Abcam/ABclonal | ab46154/A23759 |
| IL1β | Abcam | ab283818 | eNOS | Abcam | ab76198 |
| IL6 | Abcam | ab233706 | FGF1 | Abcam | ab9588 |
| CXCL8 | Abcam | ab18672 | VEGFR1 | Abcam | ab32152 |
| CXCL10 | Abcam | ab133575 | VEGFR2 | Abcam | ab39638 |
| CCL2 | Abcam | ab214819 | COL3 | ABclonal | A0817 |
| CCR7 | ABclonal | A0121 | β-actin | Abcam | ab6276 |


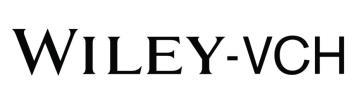


**Table S2.** The forward and reverse primer sequences of different genes for qRT-PCR analysis.

| Gene  name | Forward primer | Reverse primer |
| --- | --- | --- |
| GAPDH | TGAAGGTCGGAGTGAACGGAT | CGTTCTCAGCCTTGACCGTG |
| β-actin | GCAGAAACGAGACGAGATTG | GCAGAACTTTGGGGACTTTG |
| CD14 | GAACATTGCCCAAGCACACAC | AACTTGTGGGGACAGAGAGCA |
| TLR4 | GTGGGCTTAGAACAACTGGAAC | AGGAAATGTCAAGGTAAAGGAGG |
| MYD88 | GCCAGAAGTACATTTGGAAGCA | TGGGGCAGTAGCAGATGAAG |
| IRAK1 | AGCAGCATCTCGGAATCACC | GATGGCAACGCTGGATGGA |
| MAP2K3 | TCCTGCGGTTCCCTTACGA | TCGGCGGGGTTCTTTCTCA |
| CD68 | GGTTGGGAACTACACGTGGACTA | CCTGGGTCCTGCTTGAATCC |
| CD86 | GGACTGAGTGTCACGGTCTTTG | GATACACGCCCTTGTCCTTGA |
| TNFα | CTCTGCCTCAGCCTCTTCTCTT | ACTTGCGGGTTTGCTACTACG |
| IL1β | CCTGCGTGATGAAAGACGATAA | GGAAGACGGGCATGTACTCTGT |
| IL6 | CTGGTGGTGGCTACCGCTTT | GACATCATGGTCACACATCTCTTTC |
| CXCL8 | CCTGCTGTCTCTGACTCTTTGTG | CTGCTCAGCCCTCTTCAAGAATAT |
| CXCL10 | GGGACTAAAGGAATGCCTCTCTCTA | TCCTCTGGACCTTTCCTTGCTAA |
| CCL2 | TCAACAGCACCAAGTGTCCCA | TTGGGTTGTGGAATAAGAGGTCA |
| CCR7 | TGGTGGTGTTGACCTACATCTACTT | CGCTGAAGAAGCTGACCTTGTAG |
| FGFR1 | AGGCTACAAGGTCCGCTATGC | TGCCGTACTCGTTCTCCACAA |
| PDGFRA | GGTGTCACAATGCTGGAAGAAA | CACAGCGATGGTGACCTTCTT |
| VEGFR2 | TCCCTGCCTACCTCACCTGTTT | TTGCTGGACATCATCCCACTAAA |
| PRKCA | GCCCAAAGTGTGTGGCAAAG | CACGAACTGGGGGTTGACAT |
| ELN | TCCTGGGATTGGAGGCATTG | CAGCTCTGGCTCCGTACTTG |
| COL3A1 | CCCCGTATTATGGAGATGAACC | CCATCAGGACTAATGAGGCTTTC |
| FN1 | TTCCACACCCCAACCTTCAC | TGACCAGAAGTGCCAGGAAG |
| eNOS | TGACCCTCACCGCTACAACA | ACAATGGTCACTTTGGCGAG |
| VEGF | TTCAACGTCACCATGCAGAT | AAATGCTTTCTCCGCTCTGA |
| CD31 | CACGGTGATTCTGAACAACAAGG | TTCTATCGCCTCCTTCTTGTCC |
